# Multiple Language Use Influences Oculomotor Task Performance: Neurophysiological Evidence of a Shared Substrate between Language and Motor Control

**DOI:** 10.1371/journal.pone.0165029

**Published:** 2016-11-10

**Authors:** Karin Heidlmayr, Karine Doré-Mazars, Xavier Aparicio, Frédéric Isel

**Affiliations:** 1 Laboratory Vision Action Cognition–EA 7326, Institute of Psychology, Paris Descartes–Sorbonne Paris Cité University, Paris, France; 2 Laboratory MoDyCo–UMR 7114, CNRS, Paris Lumières–Paris Ouest Nanterre La Défense University, Paris, France; 3 Laboratory CHArt–EA 4004, Paris Est Créteil University, Paris, France; University of Akron, UNITED STATES

## Abstract

In the present electroencephalographical study, we asked to which extent executive control processes are shared by both the language and motor domain. The rationale was to examine whether executive control processes whose efficiency is reinforced by the frequent use of a second language can lead to a benefit in the control of eye movements, i.e. a non-linguistic activity. For this purpose, we administrated to 19 highly proficient late French-German bilingual participants and to a control group of 20 French monolingual participants an antisaccade task, i.e. a specific motor task involving control. In this task, an automatic saccade has to be suppressed while a voluntary eye movement in the opposite direction has to be carried out. Here, our main hypothesis is that an advantage in the antisaccade task should be observed in the bilinguals if some properties of the control processes are shared between linguistic and motor domains. ERP data revealed clear differences between bilinguals and monolinguals. Critically, we showed an increased N2 effect size in bilinguals, thought to reflect better efficiency to monitor conflict, combined with reduced effect sizes on markers reflecting inhibitory control, i.e. cue-locked positivity, the target-locked P3 and the saccade-locked presaccadic positivity (PSP). Moreover, effective connectivity analyses (dynamic causal modelling; DCM) on the neuronal source level indicated that bilinguals rely more strongly on ACC-driven control while monolinguals rely on PFC-driven control. Taken together, our combined ERP and effective connectivity findings may reflect a dynamic interplay between strengthened conflict monitoring, associated with subsequently more efficient inhibition in bilinguals. Finally, L2 proficiency and immersion experience constitute relevant factors of the language background that predict efficiency of inhibition. To conclude, the present study provided ERP and effective connectivity evidence for domain-general executive control involvement in handling multiple language use, leading to a control advantage in bilingualism.

## Introduction

### Executive control and neuroplasticity in bilingualism

Individuals need to constantly adapt to environmental constraints and neuroplasticity allows for this adaptive capacity over the lifespan [1,2]. Flexible adaptation is amongst others required in linguistic interaction, especially if an individual has acquired competence in more than one language [3,4]. In multilingualism, significant neuroplastic changes have been observed in language learning or immersion experience, both of which are highly challenging situations, in linguistic and cognitive terms [5,6]. Structural changes found with multiple language use were, e.g. grey matter volume increases with intense language interpretation studies [7], increases of grey matter density with improving L2 proficiency in immersion [8], or white matter connectivity increases with intense classroom language training [9]; for a review, see [6]. Importantly, in sustained multiple language use, not only the neurocognitive language system but also the involved control processes need to adapt in order to meet the cognitive requirements. Indeed, second-language users of a language have different control demands than native speakers, due to the reduced automaticity of the second language [10,11], the simultaneous activation of languages [12–18] and the bidirectional cross-language influences [19–21]. Therefore, psycholinguistic models of language control have to discuss the different control processes and to ask to which extent these processes are shared by different cognitive functions (language, memory, attention). A longstanding debate has opposed theories, which proposed that control processes are domain-general [4,17,22] (for some psycholinguistic theories the use of these processes is limited to low proficient bilinguals [23,24]), to others postulating that these processes are at least to a large degree domain-specific [25] (for some psycholinguistic theories this is the case for bilinguals with a high proficiency in both languages [23,24]). In the present study, we asked whether some control processes are shared by both the language and the motor domain. The rationale was to examine at the neurophysiological level whether executive control processes whose efficiency is assumed to be reinforced by the frequent use of a second language can lead to a benefit for realizing a motor task involving control, i.e. the antisaccade task.

The question on the functional architecture of executive control processes across different functions is directly related to the broader theoretical framework of embodied/grounded cognition. Embodied cognition theories feed the debate on the main question of the shared vs. distinct nature of linguistic and sensory-motor processing in handling natural language. Increasing neuroimaging and neurophysiological evidence supports the view of a distributed interactive systems account (cf. “embodied cognition” or “grounded cognition”; [26,27], e.g. the coactivation of classical language and motor regions during action word processing; [28], or the influence of word reading on motor control; [29]).

Executive functions (EF) constitute “a set of general-purpose control processes that regulate one’s thoughts and behaviors” [30]; see also [31]. It has previously been suggested, that three main executive functions can be distinguished, i.e. inhibition of prepotent responses (“inhibition”), information updating and monitoring (“updating”) and mental set shifting (“shifting”) [30,31]. On a more fine-grained level of analysis these three executive functions can be further subdivided into more specific control functions [30]. For executive control, manifold empirical evidence lends support to the theoretical accounts claiming a shared nature of (domain-general) control between cognitive domains, i.e. showing overlapping neuronal activation for linguistic and non-linguistic control [32–34], as well as improved performance in bi- and multilinguals in both linguistic and non-linguistic executive control tasks [35–41]; there is however no unanimity in this regard, for reviews, see [40,42–45]. These findings constitute major arguments in favor of models postulating domain-general control processes to be involved in the control over multiple language use.

It is important to note that the inconsistency that some studies do and others fail to show a bilingualism advantage in executive control processes may be explained by the fact that bi- or multilinguals vary on different dimensions, e.g. second language proficiency, frequency of first and second language use, or the type of interactional context (for a discussion, see [40], but also [37]), which is thought to be related to a differential recruitment of executive control processes [3,4]. As a consequence, different profiles of bilingualism may involve specific sets of control processes. Among these control processes, *conflict monitoring* performance in non-verbal tasks has been found to be improved in bilingualism in a behavioral study using the Attentional Network Task (ANT) [46], an advantage that has been found in a neurophysiological study to be especially strong in bilinguals with good control over their language switches [47], or in bilinguals with high second language proficiency, as observed in a neurophysiological study using a saccadic countermanding task [48]. The saccadic countermanding task is used to examine the neural response of movement initiation and suppression, i.e. the response adjustments during action control. This task involves the inhibition of an *initiated* saccade and the voluntary redirection of the gaze to a new location [48,49]. A behavioral advantage in *response inhibition* as well as task *switching* performance was found in a modified antisaccade task in older but not in younger adult bilinguals [50]. Using the same task, a *switching* advantage was also observed in bilingual children [51]. These data collected from different age groups suggest that (1) activity-dependent long-term effects on executive function capacity vary over the lifespan, and (2) activity-dependent improvements are more likely to occur in age groups with generally lower than peak executive function (EF) capacity, i.e. children and older adults. In a non-verbal task switching paradigm, Prior and MacWhinney [41] found a behavioral bilingual advantage for switching and Prior and Gollan [52] found that the benefit was strongest if bilinguals reported a high frequency of daily language switching. Concerning *vector inversion/movement initiation*, to our knowledge there are no previous studies that have tackled the question of an influence of bilingualism.

More recently, intermediate theoretical positions postulating a hybrid neural architecture of executive control and its interaction with the language domain have emerged, which try to account more specifically for the relation between domain-general and domain-specific processes. One account postulates, that domain-general executive control is involved in language processing, i.e. that there is an interaction between the neurocognitive networks of domain-general executive control and of language [53]. Based on network neuroscience, it is claimed that in neurocognitive networks, e.g. the language network, there is a distinction between a domain-specific network core and domain-general periphery, which is shared with other neurocognitive networks, e.g. the domain-general control network [53]. This perspective may help identifying domain-specific and domain-general processes in language processing [53]. Similarly, however for control in non-linguistic domains, one account considers that there is an interplay between both domain-general control processes, that are shared across cognitive and motor domains, and domain-specific control processes, that are specific to the control challenges in a particular cognitive or motor domain [25]. Multiple findings on language and non-linguistic control, showing evidence for an overlap across domains for some control processes while not for others support these theoretical accounts [54,55]. To date, there is no consensus on whether executive control processes are partially or fully shared between different domains (linguistic, non-linguistic, motor).

The goal of the present electroencephalographic study was to investigate whether executive control processes whose efficiency can be increased by the use of multiple languages are also involved in nonlinguistic activities such as the control of eye movements. For this purpose, we administrated to highly proficient French-German bilingual participants and to a control group of French monolingual participants an antisaccade task, i.e. a task involving motor control, in which an automatic saccade needs to be suppressed and a voluntary eye movement in the opposite direction needs to be carried out. The main hypothesis of our study is that a behavioral and/or a neurophysiological advantage of bilingualism should be observed if the control processes involved in the antisaccade task are shared between linguistic and motor domains. Else, no bilingualism advantage should be expected with the antisaccade task. Critically, we examined whether individuals who use more than one language on a daily basis (‘bilinguals’) show a better performance in controlling predominant, automatic responses in a non-linguistic motor task, i.e. the antisaccade task, than individuals who use solely one language (‘monolinguals’). To our knowledge, it is the first time that the neuronal underpinnings of oculomotor control processes and the time course of their activation were investigated in relation to language control. The paradigm chosen for the present study was a version of the antisaccade task in which the involvement of the processes of *conflict monitoring*, *response inhibition*, *vector inversion/movement initiation* as well as *switching* (task engagement and disengagement, attentional shifting) can be studied. Moreover, the role of the neuronal regions relevant for these control processes, mainly the anterior cingulate cortex (ACC) and the prefrontal cortex (PFC), as well as their interaction were examined in order to compare neuronal dynamics underlying control processes between the two groups.

### The antisaccade task: control processes and neuroanatomical regions

The saccade task [56] is a task that allows for studying the voluntary control of action [57]. Participants are instructed to carry out either an automatic eye movement towards a visual target (prosaccade) or suppress this automatic eye movement and effectuate a saccade into the opposite direction (antisaccade), which depends on the color of the instructional cue preceding the target stimulus. Miyake and Friedman [30] classify the antisaccade task as a representative task to study *inhibition*, defined as the “deliberate overriding of dominant or prepotent responses”; more specifically the antisaccade task may require *response inhibition* [58]; for theoretical accounts claiming a separation between *response inhibition* and *interference suppression*, see [59,60]; see however [61]. Munoz and Everling [57] claimed that the antisaccade task does not only require response inhibition of the automatic prosaccade but also *vector inversion* (i.e. direction inversion), that is the stimulus vector must be inverted into the saccade vector in order to initiate a voluntary antisaccade (see also [62]). Furthermore, it has been suggested that *conflict monitoring* is also a relevant control process for successful antisaccade performance [63,64]. In general, conflict monitoring has been defined as the processes of monitoring for the occurrence of conflict in information processing and is on the evaluative side of cognitive control. In the antisaccade task, conflict monitoring is required because the requirement to look away from a visual stimulus creates a conflict between two opposing saccade commands, an automatic (sensory-driven) saccade toward the stimulus and a voluntary (internally driven) saccade away from the stimulus [65]. Conflict monitoring serves to translate the occurrence of conflict into compensatory adjustments in control, i.e. the conflict monitoring system evaluates the levels of conflict and communicates this information to systems responsible for control implementation [63]. In addition to *response inhibition*, *vector inversion* and *conflict monitoring*, control adjustment is also required in the transition between trials of different conditions, i.e. *switching*-related control processes, in the antisaccade task. In linguistic and non-linguistic tasks, the ability to *switch* between different task-sets reflects the flexibility and ease of transitioning to new task-set representations [30]. The switching process involves task disengagement, task engagement, suppression of previous task sets [4,66,67], overcoming of inhibition and attentional shifting [68]. When the direction of switch is from a more difficult towards an easier task, previously applied sustained inhibition needs to be overcome, which is not the case in switching from the easier to the more difficult task, producing the robustly observed asymmetrical switching cost [68]; for a review and alternative accounts to explain asymmetrical switching costs, see [67].

**Fig 1:**
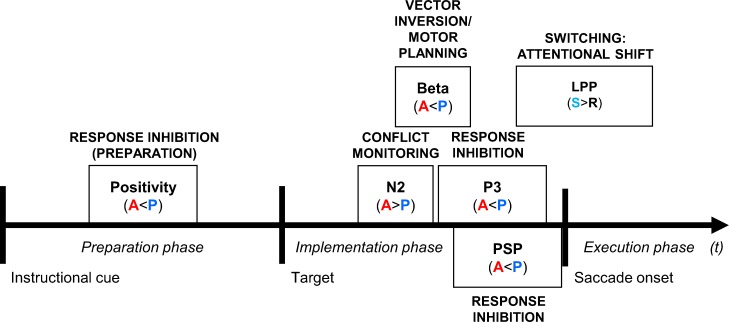
ERP components. Overview of the different ERP components and the oscillatory marker reported in the preparation, implementation and execution phases of saccadic eye movements in cue-locked, target-locked and saccade-locked epochs. PSP: presaccadic positivity; LPP: late parietal positivity; A: antisaccade; P: prosaccade.

At the neuroanatomical level, the suppression and/or generation of saccadic eye movements involves activation in a number of cortical and subcortical structures, i.e. the dorsolateral prefrontal cortex (PFC), the anterior cingulate cortex (ACC), the lateral intraparietal area (LIP), the supplementary eye fields (SEF), the frontal eye fields (FEF), the superior colliculus (SC), the substantia nigra pars reticulata (SNpr) [57], the striatum [65,69] and the thalamus; for reviews, see [57,69,70]. During antisaccades, the automatic activation of saccade neurons contralateral to the visual target needs to be inhibited while saccadic activity ipsilateral to the stimulus (contralateral to the target movement) is required. The *inhibition* of saccade neurons is thought to be carried out by fixation neurons and interneurons in the FEF and SC, which receive the information to do so probably from the PFC, the SEF, or the SNpr. The neuronal underpinnings of *vector inversion*, which is required for carrying out correct antisaccades beside *response inhibition*, are not yet very well understood but there is evidence that the LIP–which is at the interface between sensory and motor processing–and the FEF play a role in this process. Moreover, the anterior cingulate cortex (ACC) has been found to play a role in reflexive saccade suppression [70,71] and is thought to be active during *conflict monitoring* processes involved in antisaccade trials [63,70,72,73]. Once a pro- or antisaccade is initiated, fixation neurons in the FEF and SC cease to fire and there is a buildup of activity in saccade neurons. Control-related ERPs and oscillatory activity in the antisaccade task, thought to reflect *conflict monitoring*, *response inhibition*, *vector inversion/motor planning* and *switching*, will be presented as follows and the ERP markers are schematized in Fig 1.

### ERP and oscillatory markers of control processes in an antisaccade task

The above-mentioned control processes in an antisaccade task are associated with distinct ERP and oscillatory markers. For presenting these processes and their associated neurophysiological markers, we decided to follow their hypothesized chronological order: (1) *conflict monitoring*, (2) *response inhibition*, (3) *vector inversion/motor planning* and lastly, however concerning the transition between trials: (4) *switching*-related processes. Moreover, for each component, the phase in which it occurs, i.e. the ‘preparation’ (cue-locked), ‘implementation’ (target-locked) and ‘execution’ (saccade-locked), will be indicated. *Conflict monitoring* has previously been ascribed to a stimulus-locked fronto-central N2 component that is larger in the condition requiring control (i.e. antisaccade or nogo condition) as compared to a control condition (i.e. prosaccade or go condition; [74–76]; for a different view on the N2 in the antisaccade task, see [68]). The anterior cingulate cortex (ACC) is considered as a principal neuronal generator of the fronto-central N2 associated with conflict monitoring [72,77]. The process of *response inhibition* of the automatic prosaccade in an antisaccade task is thought to be reflected by a target-locked parietal positivity at around 300 ms post-target onset (P3) which was shown to be reduced in anti- compared to prosaccades [68] and the prefrontal cortex (PFC) has been found as one of its main neuronal generators [78]. Although such a reduction of the P3 amplitude for a task involving inhibition can sound counterintuitive, however, it makes sense when, as it was suggested, the P3 modulation may reflect the decision to withhold a response [79]. Reduced P3 amplitudes have also been observed in the Eriksen Flanker task [80], the go/no-go task [79], or in a partially incongruent categorization task [78] and the processes underlying the reduction of the P3 amplitude may not be located in one single neuronal generator but may consist of a combination of the activity in inhibitory control regions and/or the outcome of high-level inhibitory control at target sites, e.g. the motor cortex [75]. Given that the increased N2 and reduced P3 frequently occur together they are frequently also considered as an ‘N2/P3 complex’ [68,81,82]. Another inhibitory component, but occurring during the preparation stage, is the frontal cue-locked positivity around 200–300 ms post-cue onset that is smaller in anti- than prosaccades [68] which probably reflects preparatory processes for the decision to withhold an automatic response. A third component that has been suggested to reflect inhibitory processes occurs during the saccade execution stage, i.e. a saccade-locked central presaccadic positivity (PSP) over a period of 250–50 ms prior to saccade onset that is smaller before anti- than prosaccades [83,84]. *Vector inversion and motor planning* have been found to be reflected by a fronto-central and occipital power decrease in the beta band (13–26 Hz) [85,86]. As for *switching*-related components, a target-locked late parietal positivity (LPP) for switch vs. repetition trials at approximately 500–600 ms has been found to be larger for switch as compared to repetition trials, for both antisaccades and prosaccades, and is thought to reflect attentional shifting to the relevant task [68].

### The present study

At present, there is behavioral and neuroimaging evidence in the literature that multiple language use involves domain-general control mechanisms which are also involved in non-linguistic control. Different evidence, however, gives support to hybrid accounts postulating that there are partially domain-general and partially language domain-specific control processes involved. The present study investigates the relation between language and oculomotricity and particularly asks whether bilinguals show a benefit in realizing a motor task involving control. The contribution of our study is to examine the neurodynamics of different control processes and subprocesses involved in an antisaccade task at the preparation (cue-locked), implementation (target-locked) and execution (saccade-locked) stages. EEG is a technique that allows to track phases of neuronal activation on a temporally fine-grained scale and therefore is a particularly useful technique for disentangling activity associated with different neurocognitive control processes and subprocesses. This should allow us to disentangle the processes for which an impact of bilingualism can occur. Moreover, source localization analyses will enable to identify neuronal generators associated with EEG markers thought to reflect these processes. Finally, by conducting DCM (dynamic causal modelling) analyses we aim at identifying effective connectivity dynamics at the network level between these neuronal regions. Effective connectivity concerns how activity in one brain region influences activity in another region [87]. In the present study, the investigation of effective connectivity will allow for learning more about the interplay of ACC and PFC in conflict monitoring and response inhibition processes in bilinguals and monolinguals. Note that despite the attested role of subcortical structures and of fronto-subcortical connections in bilingualism [88,89] as well as in saccade control [57,65,69] these sources were not included in our analyses due to the low precision for subcortical structures in source reconstruction analyses for ERPs.

In the present study, we aim to test if an impact of bilingualism on control performance can be observed in non-verbal oculomotor task. If control processes that are supposed to be involved in and trained by multiple language use show higher efficiency in a non-linguistic task, this can be considered as evidence in favor of accounts claiming domain-generality of control processes. If however, a more mitigated picture emerges, with only some processes being influenced by bilingualism while others are not, this can be taken as evidence supporting hybrid accounts claiming partially domain-general and partially domain-specific processes to be involved in language control. Moreover, in the present study, we aimed at investigating the predictive power of, e.g. second language proficiency, immersion experience, frequency of second language use, and frequency of language switching, on eye movement control capacity. By doing so, we aimed at giving a more fine-grained account of the multifaceted nature of bilingualism than has been previously done.

For behavioral measures we expect to find longer saccade latencies and higher error rates for antisaccades than for prosaccades. Saccade latencies were measured as the duration from target onset until saccade onset and are given in milliseconds. As erroneous responses were considered expected saccade direction errors as well as saccade omissions (absence of saccade initiation) and are given as a percentage. Moreover, saccade latencies are predicted to be longer for switch compared to repetition trials. This transition effect (switching effect) is predicted to be larger for prosaccades than for antisaccades (AP > PP; **>**; PA > AA). Both, the saccade task effect (‘Antisaccade effect’) and the transition effect (switching effect) are expected to be larger for monolinguals than for bilinguals. At the neurophysiological level, a bilingual advantage for conflict monitoring should be reflected by a larger N2 effect size. This, advantage is expected to be modulated by the self-reported degree of control over and the frequency of daily language switching. A bilingual advantage for response inhibition should be reflected by smaller effect sizes of the cue-locked positivity, the P3 and PSP in bilinguals as compared to monolinguals. The processes of *vector inversion and motor planning* should be associated with a target-locked fronto-central and occipital beta (13-26Hz) power decrease in anti- compared to prosaccades. Finally, higher switching capacity should be reflected by smaller LPP effect sizes in bilinguals, reinforced in bilingual individuals with better controlled and more frequent daily language switching.

## Methods

Approval for the study was given by the local Ethics Committee (*Conseil d’évaluation éthique pour les recherches en santé* at Paris Descartes–Sorbonne Paris Cité University) and the participants gave their informed written consent prior to participating in the study.

### Participants

Forty right-handed participants (Edinburgh Handedness Inventory) were recruited. By their own account, participants had no history of current or past neurological or psychiatric diseases, they had normal or corrected-to-normal vision and normal color vision. Twenty of the participants were native speakers of French (L1) and highly proficient second language speakers of German (L2). One bilingual participant was excluded from the analyses due to a high degree of motor artifact contamination of the recorded EEG. The second group of 20 participants were native speakers of French (L1) with reduced use of other languages than their mother tongue (Frequency of daily non-native language use: 1.4 ± 1.2%). All participants were selected after having completed a language history questionnaire. Data on linguistic and environmental background measures can be found in Table 1. The average age did not differ between bilinguals (*n* = 19; 12 females, 7 males; 22.5 ± 2.6 years, range 19–30) and monolinguals (*n* = 20; 10 females, 10 males; 23.8 ± 5.1 years, range 19–37; *F* < 1). The two groups were matched for socio-economic status using the educational and professional status, i.e. all bilingual and monolingual participants were students or young university graduates. Bilinguals were late learners of German who studied the language at secondary school in France as their first non-native language (L2; Age of acquisition (AoA): 10.4 ± 0.8 years). They all had a regular use of their L2 (L2 daily frequency of use: 21.2 ± 16.9%) and 84% of the bilingual participants (16 individuals) also frequently used an additional L3 (L3 daily frequency of use: 6.5 ± 6.4%). The bilinguals’ self-reported proficiency of 1.3 ± 0.6 [scale: 1: good– 5: poor] was high, which was also confirmed by the percentage of correct responses on a standardized test of German as a foreign language (*DAF–Deutsch als Fremdsprache*): 84.1 ± 7.4%.

**Table 1:** Background data. Linguistic and environmental background measures as assessed by a questionnaire are reported in the table. The mean and standard deviation (SD) is indicated for each category.

	Bilinguals		Monolinguals		*p*
	(*n* = 19)		(*n* = 20)		
	Mean	(SD)	Mean	(SD)	
Age [years]	22.5	(2.6)	23.8	(5.1)	ns
Self-rated proficiency L2 [1: good—5: poor]	1.3	(0.6)	2.7	(1.1)	< .001
Self-rated proficiency L3 [1: good—5: poor]	2.1	(1.0)	3.8	(0.9)	< .001
Frequency of L1 use [%]	71.0	(21.6)	98.6	(1.2)	< .001
Frequency of L2 use [%]	21.2	(16.9)	1.3	(1.2)	< .001
Frequency of L3 use [%]	6.5	(6.4)	0.0	(0.2)	< .001
AoA L2 [years]	10.4	(0.8)	10.6	(0.9)	ns
AoA L3 [years]	12.5	(1.0)	12.7	(0.9)	ns
Immersion in L2 environment [years]	1.4	(0.9)	—	—	—
Age of immersion [years]	18.8	(2.8)	—	—	—
Distance of immersion [years]	2.1	(2.5)	—	—	—
L2 proficiency: Grammar [%]	93.3	(4.8)	—	—	—
L2 proficiency: Communication [%]	92.7	(5.2)	—	—	—
L2 proficiency: Production [%]	66.3	(15.1)	—	—	—
L2 proficiency: Total [%]	84.1	(7.4)	—	—	—
Vid/Comp games [hour/week]	2.0	(4.8)	0.9	(1.4)	ns
Sport practice—high coordination [hour/week]	2.0	(3.1)	0.7	(1.2)	ns
Music practice [hour/week]	0.7	(1.6)	0.5	(0.9)	ns

AoA, Age of acquisition; L2 proficiency: Communication, Test of the participant’s understanding of meaningful conversational interaction; L2 proficiency: Grammar, Test of the participant’s grammatical competence; L2 proficiency: Production, Test of the lexical and syntactic accuracy of the participant’s written production; Vid/Comp games, Video and Computer games.

### Stimuli and Procedure

Stimuli were displayed on an Iiyama HM240DT monitor with a refresh rate of 160 Hz and a resolution of 800×600 pixels. The experimental sessions took place in a dimly lit room. Participants were seated 22 inches away from the screen and their head kept stable with a chin and forehead rest. Eye movements were recorded with an Eyelink® 1000 (SR Research, Ontario, Canada), with a temporal resolution of 1000 Hz, and a spatial resolution of 0.15°. Viewing was binocular but only movements of the right eye were monitored. Each session began with a 9 points calibration over the entire screen. Before each trial, central fixation was checked and compared to the calibration. If the distance between the fixation check and the calibration was greater than 0.75°, fixation was refused and the trial was reinitiated. When successful calibration was detected, the trial began. Online saccade detection corresponded to above-threshold velocity (30°/s) and acceleration (8000°/s^2^).

**Fig 2:**
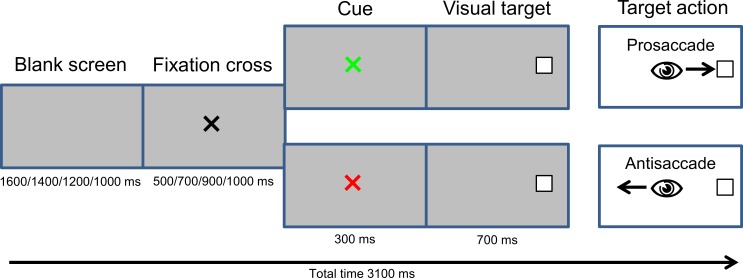
Timing of a prosaccade and an antisaccade trial.

Our experimental design was taken from the one used by Mueller et al. [68]. Each trial began with a blank screen, followed by a 1.5° x 1.5° black fixation cross presented in the center of the screen on a grey background. The combination of blank screen and fixation cross lasted 2100 ms with four different timings (1600+500 ms; 1400+700 ms; 1200+900 ms; 100+ 1100 ms respectively) randomized from trial to trial (see Fig 2), in order to avoid anticipation of cue onset. The fixation cross then turned into two different cues, a green or a red cross presented during 300 ms on the screen. The white target box (1.5° x 1.5°) then appeared on either the left or the right side of the screen, and was displayed for 700 ms on two possible eccentricities (6° and 10°) randomized across trials.

Participants were instructed to make an eye movement towards the target box (prosaccade trial) if the cue was green, and an eye movement away from the target box, in a mirror symmetric location (antisaccade trial) when the cue was red. They were instructed to hold their fixation until the disappearance of the target box, and then, look back to the center of the screen. The experimental session (768 trials) was divided into three parts. In the first part of the experiment (pre), participants monitored a single task session of 96 prosaccade trials, and then 96 antisaccade trials (the order was counterbalanced across participants of each group). In the second part (mixed task session), they monitored 384 trials with pro- and antisaccade trials mixed. Then, in the third and last part (post) they monitored again a single task session of 96 prosaccade trials and a single task session of 96 antisaccade trials (in the same order as the first part). Before each task (prosaccades or antisaccades single task sessions or mixed task sessions), a block of training was proposed in order to familiarize the participants with the task. A short break was allowed to the participants between each block. All together (including single task and mixed task sessions), there was the same number of pro- and antisaccades (384). In the mixed task session, there were as many switches (defined as when the current saccade task differed from the task in the previous trial) as repetition trials (defined as when the previous saccade task was identical to the saccade task in the current trial). These dyads, taking into account *n*-1 trials, were used in order to examine the switching-related processes in comparing prosaccades and antisaccades as a function of repetition and switching trials in the mixed task session. The second trial of a dyad was considered as the target element for which the influence of the preceding trial was aimed to be studied. In total, 96 trials per dyad type (PP, AP, AA, PA) were implemented over the total of 12 mixed blocks. To set up the lists of 32 dyads per mixed block, we applied amongst others the constraint that the same type of saccade was presented no more than three times in a row. For statistical analyses, only those dyads with accurate performance on *n* as well as *n*-1 trials were taken into consideration.

### Behavioral data analysis

Saccades with latencies below 50 ms were considered as early starts and removed from the analysis, as well as latencies above 500 ms, and trials containing eye blinks. Trials with latencies exceeding the threshold of mean ± 2 SD per condition and participant were considered as outliers and hence excluded from the analysis. Overall, 5.8 ± 1.6% of outlier trials were removed (single and mixed task blocks collapsed), which did not differ between saccade tasks (*p* > .05) or groups (*F* < 1). We averaged the saccade latencies for correct answers (*i*.*e*. correct saccade direction in regard to instructions) with data from both eccentricities (6° and 10°) and from both target locations (left and right). A trial was considered erroneous when the participant carried out a prosaccade if the instructional cue indicated an antisaccade trial (red cue), and if an antisaccade was carried out when the cue indicated a prosaccade (green cue). Error rates were averaged for both eccentricities and target locations.

Statistical analyses were carried out with SPSS 17.0. For analysis of the behavioral measures (saccade latencies and error rate), a three-way repeated measures ANOVA with Group as a between-subjects factor (monolingual vs. bilingual), and Saccade task (prosaccade vs. antisaccade) and Block type (mixed task blocks vs. single task blocks) as within-subjects factors was conducted. Planned comparisons were made to examine task effects for each Group. Moreover, a three-way repeated measures ANOVA with Group as a between-subjects factor (monolingual vs. bilingual), and Saccade task (prosaccade vs. antisaccade) and Transition (switch vs. repetition) as within-subjects factors was performed to examine switching-related performance on the two behavioral measures (saccade latencies and error rates).

### EEG acquisition and preprocessing

EEG preprocessing has been conducted using *Brain Vision Analyzer 2*.*1*.*0* (*Brain Products*). EEG was recorded from 33 channels placed according to the international 10–20 system (Fp1, Fp2, F7, F3, Fz, F4, F8, FC5, FC1, FCz, FC2, FC6, T7, C3, Cz, C4, T8, TP9, CP5, CP1, CP2, CP6, TP10, P7, P3, Pz, P4, P8, PO9, O1, Oz, O2, PO10), mounted in an elastic cap (*ActiCap*, *Brain Products*) and recorded with the *Brain Vision Recorder*, *Brain Products*. All channels were referenced online against FCz. For data analysis, channels were re-referenced to an average reference after having removed any bad channels. Electrode impedances were kept below 25 kΩ. Data were recorded at a sampling rate of 1000 Hz. An online band-pass filter of 0.01–100 Hz was used. Then, the data were filtered offline (IIR-Butterworth filter) with a high-pass filter at 0.05 Hz (slope 12 dB/octave) and a notch filter for 50 Hz (slope 24 dB/octave). On the continuous data, automatic artifact detection for non-ocular artifacts was conducted (Maximum amplitude difference in interval of 200 ms: 300 μV, maximum gradient voltage step: 50 μV/ms, lowest allowed activity in interval of 100 ms: 0.5 μV). Then, in order to remove artifacts from horizontal eye movements or from eye blinks from the continuous EEG signal, an ocular correction ICA (unbiased extended Infomax) was run using Fp1 as a VEOG channel and F7 and F8 as HEOG channels. Blinks were detected using the Value Trigger Algorithm (Blink trigger value: 97%, blink correlation trigger: 70%). The ocular correction ICA was applied using the whole data. Components relevant for vertical and horizontal activity were identified based on the relative variance in the respective channel(s) and the percentage of variance to delete was set to 30%. For further analyses, only trials on which the participants carried out a correct oculomotor response and which were not contaminated by anticipatory eye movements (saccade latencies faster than 50 ms), saccade latencies of more than 500 ms (indicating that the participant did not correctly follow the instructions) or other movement artifacts, were included. The continuous EEG was segmented into epochs relative to three events: (1) cue-locked segments were segmented into epochs from 500 ms pre-cue until 1300 ms post-cue onset, (2) target-locked segments were segmented into epochs from 800 ms pre-target until 1000 ms post-target onset and (3) saccade-locked segments were segmented into epochs from 1100 ms pre-saccade until 700 ms post-saccade onset.

On the percentage of trials included in ERP analyses after preprocessing and data cleaning we conducted a three-way repeated measures ANOVA with Group as a between-subjects factor (monolingual vs. bilingual), and Saccade task (prosaccade vs. antisaccade) and Block type (mixed task blocks vs. single task blocks) as within-subjects factors. There was a main effect of Saccade task (*F*(1, 37) = 53.31, MSE = 183.18, *p* < .001, *η*^2^_p_ = .590), indicating that the percentage of included trials was higher in prosaccades (70.2 ± 14.7%) than antisaccades (54.4 ± 15.7%). Moreover, there was a Saccade task by Block type interaction (*F*(1, 37) = 43.99, MSE = 45.34, *p* < .001, *η*^2^_p_ = .543), reflecting that for prosaccades a higher percentage of trials was kept in mixed (73.4 ± 14.7%) compared to single block sessions (67.0 ± 16.5%; *p* < .001), while in antisaccades a smaller percentage was kept in mixed (50.4 ± 18.1%) than single task sessions (58.4 ± 15.2%; *p* < .001). There was no other main effect or interaction.

### ERP analysis

ERP preprocessing and analyses have been conducted using *Brain Vision Analyzer 2*.*1*.*0* (*Brain Products*) and *EEGLAB* toolbox (version 13.2.2) [90] for MATLAB (version 7.12.0, R2011a) and SPSS 17.0. Event-related brain potentials (ERPs) were computed for each participant in each experimental condition for cue-, target- and saccade-locked epochs. Cue-locked segments were baseline corrected with the baseline set from 100 ms pre-cue onset until cue onset. Target-locked segments were baseline corrected with the baseline set from 400 ms to 300 ms pre-target onset (which is equivalent to the 100 ms baseline before cue onset). Saccade-locked segments were baseline corrected with a baseline set from 700 ms to 600 ms pre-saccade onset (which covers a time window that is on average before cue-onset). Then, in each experimental condition, the ERP activity was averaged over trials and over participants (i.e. grand average ERP). Statistical analyses of the ERP effects were performed for cue-, target- and saccade-locked ERPs in selected time windows based on previous studies [68,83,84] and adjusted by visual inspection of the grand averages (Preparation phase: Cue-locked positivity: Cue-locked 150–250 ms; Implementation phase: N2: target-locked 160–200 ms, P3: target-locked 200–400 ms, Late parietal positivity (LPP): target-locked: Antisaccades: 400–650 ms, Prosaccades: 400–550 ms; Execution phase: Presaccadic positivity (PSP): saccade-locked -250 to -50 ms). Mean amplitudes were calculated for each time window. All analyses were quantified using the multivariate approach to repeated measurement and followed a hierarchical analysis schema. In order to allow for an examination of hemispheric differences, the data recorded at the midline electrode sites were treated separately from the data recorded from lateral recording sites. Four-way repeated measures ANOVAs were conducted for the analyses for the lateral electrodes, including the within-subjects factors Saccade task (prosaccade vs. antisaccade) as well as two topographical variables Region (anterior vs. posterior) and Hemisphere (left vs. right) and the between-subjects factor Group (monolingual vs. bilingual). Four regions of interest (ROIs) resulting from a complete crossing of the Region and Hemisphere variables were defined: left anterior (F3, FC1, FC5), right anterior (F4, FC2, FC6), left posterior (CP5, CP1, P3), and right posterior (CP6, CP2, P4). The data from the midline electrodes were analyzed with a three-way repeated measures ANOVA including the within-subjects factors Saccade task (prosaccade vs. antisaccade) as well as Electrode (Fz, Cz, Pz) and the between-subjects factor Group (monolingual vs. bilingual). Note that for one bilingual and for one monolingual participant the Fz electrode provided noisy data and these two participants were consequently excluded from the analyses on midline electrodes, which explains the reduced degrees of freedom in these analyses. To investigate switching-related activity, separate analyses were conducted for antisaccades and prosaccades, respectively. Four-way repeated measures ANOVAs were conducted for the analyses on the lateral electrodes, including the within-subjects factors Transition (switch vs. repetition) as well as two topographical variables Region (anterior vs. posterior) and Hemisphere (left vs. right) and the between-subjects factor Group (monolingual vs. bilingual). Three-way repeated measures ANOVAs were conducted for the analysis of data from the midline electrodes, including the within-subjects factors Transition (switch vs. repetition) as well as Electrode (Fz, Cz, Pz) and the between-subjects factor Group (monolingual vs. bilingual). The dependent variable was the voltage amplitude [μV] averaged over the relevant interval of each ERP component of interest. The Greenhouse–Geisser correction [91] was applied when evaluating effects with more than one degree of freedom in the numerator. Post-hoc pairwise comparisons at single electrode sites were performed using a modified Bonferroni procedure [92]. A significance level of 0.05 was used for all statistical tests and only significant results are reported. For visual presentation of the midline electrodes in detail figures, curves were smoothed (1:50 points).

### Dynamic causal modelling (DCM) analysis

**Fig 3:**
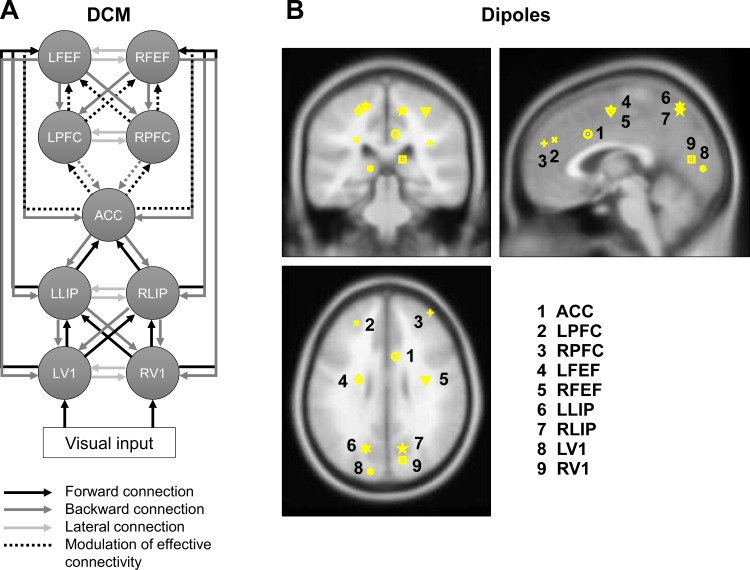
Effective connectivity model tested in a DCM analysis and equivalent current dipole locations. **A** The neuronal sources in the model are connected with forward (black), backward (dark grey) or lateral (light grey) connections. Connections that are modelled to vary between experimental conditions are depicted with dotted lines. Connections between V1 and FEF as well as LIP and FEF also connect to the contralateral side but are depicted only for the ipsilateral side for the sake of clarity of the figure. **B** Locations of the equivalent current dipoles included in the model are depicted in an MRI of a standard brain in MNI space. ACC, Anterior cingulate cortex; LFEF/RFEF, left and right frontal eye field; LLIP/RLIP, left and right lateral intraparietal area; LPFC/RPFC, left and right prefrontal cortex; LV1/RV1, left and right primary visual cortex.

The dynamic causal modelling (DCM) analysis was conducted in SPM12 (Wellcome Trust Centre for Neuroimaging, London, UK) and SPSS 17.0. DCM is a method that allows to test hypotheses of dynamics in a neuronal network which need to be defined as effective connectivity models. Effective connectivity allows us to examine how activity in one brain region influences activity in another region [87]. Here, based on (1) previous models of both saccade control [93] and the antisaccade task [57], as well as (2) fMRI evidence with an antisaccade task [94–96] and (3) TMS-MEG evidence of frontal top-down control on occipito-parietal excitability [97], a highly plausible effective connectivity model was created (Fig 3) and tested for effective connectivity differences between groups. Note that source reconstruction analyses for ERPs have sufficient precision only for cortical sources and consequently our modeling did not include any subcortical structures, despite their evident role in saccade control. Nine cortical sources, modeled as equivalent current dipoles (ECDs), were included in our effective connectivity model: left and right primary visual cortex (LV1, RV1), left and right lateral intraparietal area (LLIP, RLIP), anterior cingulate cortex (ACC), left and right prefrontal cortex (LPFC, RPFC), left and right frontal eye field (LFEF, RFEF). Table 2 presents the MNI coordinates and Fig 3B the locations of these cortical neuronal generators taken from two fMRI studies on an antisaccade paradigm, i.e. Ford [96] and Aichert, et al. [94], and transformed from Talairach to MNI space using the tal2mni tool (http://imaging.mrc-cbu.cam.ac.uk/imaging/MniTalairach). The source locations were specified while the dipole orientation parameters were left free and were individually adjusted during the model inversion process. During the model inversion process, DCM optimizes for each participant the information provided concerning the electromagnetic forward model and the neuronal sources (i.e. the number, locations and connections of the neuronal sources), aiming at minimizing free energy [98]. As forward model for the ERP data of the present study, the boundary element model (BEM) implemented in SPM12 was used.

**Table 2:** Coordinates of neuronal sources used in the DCM analysis. Source coordinates have been taken from *Ford **[96]** and ^Δ^Aichert et al. **[94]** and were transformed from Talairach to MNI space using the tal2mni tool (http://imaging.mrc-cbu.cam.ac.uk/imaging/MniTalairach).

Brain region	Abbrev.	BA	MNI coord.			Cognitive processes
			***x***	***y***	***z***	
Left primary visual cortex	LV1	17	-14	-88	4^**Δ**^	visual stimulus processing
Right primary visual cortex	RV1	17	14	-78	12^**Δ**^	visual stimulus processing
Left lateral intraparietal area	LLIP	7	-18	-68	58^**Δ**^	sensory-motor transformation
Right lateral intraparietal area	RLIP	7	14	-68	54^**Δ**^	sensory-motor transformation
Anterior cingulate cortex	ACC	32	8	12	34*	cognitive control; attention, motor modulation, response selection
Left prefrontal cortex	LPFC	10	-26	41	29*	cognitive control
Right prefrontal cortex	RPFC	10	38	50	26^**Δ**^	cognitive control
Left superior frontal gyrus (left frontal eye field)	LFEF	6	-24	-8	54^**Δ**^	eye movement control, inhibition
Right medial frontal gyrus (right frontal eye field)	RFEF	6	34	-8	54^**Δ**^	eye movement control, inhibition

Abbrev., Abbreviation; BA, Brodmann area; Coord., coordinates.

The DCM model was designed as follows: Given that our paradigm involved visual stimulation, the left and right primary visual cortex were defined as input regions. The time window used for model adjustment started with cue-onset and ended at 400 ms after target-onset and consequently modeled source activity underlying the cue-locked positivity, the N2, the P3 and the PSP effects. The visual stimulation by the presentation of the cue was modeled at starting at 0 ms of the time window and reaching the input region 64 ms after cue onset (this delay is an approximation of when the stimulation activates the cortical areas of the model, see [99]). The target onset was modeled at time point 300, reaching the input region 64 ms after target onset. The following DCM was modeled: The visual input (cue, target) entered bilaterally to the primary visual cortex (LV1/RV1), which were connected to ipsi- and contralateral lateral intraparietal areas (LLIP/RLIP), which again were connected to the anterior cingulate cortex (ACC) in the frontal lobe. The ACC was bilaterally connected to the prefrontal cortex (LPFC/RPFC), which again had connections to ipsi- and contralateral frontal eye fields (LFEF/RFEF). Lateral connections were assumed between the left and right V1, left and right LIP, left and right PFC and left and right FEF. All connections were reciprocal and connected with the ipsi- as well as the contralateral site.

Here, DCM analyses were conducted for pro- and antisaccades issued from mixed task blocks. In the DCM model, the connections that are hypothesized to be modulated by experimental condition (‘modulatory connections’), i.e. Saccade task (prosaccade vs. antisaccade), are those from ACC to PFC and backward from PFC to ACC, from PFC to FEF and from ACC to FEF. After inversion of the model on each subject’s data set, the values of the effective connectivity in each modulatory connection, i.e. connections between neuronal sources that were allowed to vary between prosaccades vs. antisaccades, were extracted for each participant. The value for each modulatory effective connection was subsequently submitted to an independent samples t-test with Group as a between-subjects factor.

### Correlation analyses with language background factors

Finally, the information on the individual language background of the bilingual participants in our study were put into relation with the experimental measures on which group differences were observed. These analyses were done using SPSS 17.0. Most of the language background factors included in our language background questionnaire, e.g. language switching experience, immersion in L2 environment, L2 proficiency, and the motivation to improve L2 proficiency, were addressed with more than one question or test in order to obtain a precise picture. For each language background factor, the relevant sub-scores in the questionnaire were identified and included in the principle component analysis (PCA) if the responses showed reasonable variance, i.e. for instance were sub-scores not included if all participants responded with the highest score on the scale. The included variables were standardized using a correlation matrix, an orthogonal rotation method (Varimax with Kaiser normalization) was applied and the principle component analysis was constrained to extract only one component. The language background factors and the corresponding questions are presented in Table 3. Correlations involving these five language background factors, i.e. language switching experience, immersion in L2 environment, L2 proficiency, motivation to improve L2 proficiency, and the frequency of daily L2 use [%] and the ERP effect sizes and DCM modulatory connections for which group differences had been found, were conducted for the bilingual group.

**Table 3:** Language background factors. One principal component for each of the language background factors of interest was extracted from the responses to the corresponding questions in the questionnaire.

Factor		Information assessed in questionnaire	Unit
Language switching experience	(1)	Frequency of daily switching in general	[h/day]
	(2)	Frequency of daily switching in professional activity	[h/day]
	(3)	Frequency of daily switching at week-ends	[h/day]
	(4)	Frequency of daily switching during leisure time	[h/day]
	(5)	Automaticity of language switching	10-point scale [10: highly automatic—1: requiring a lot of control]
Immersion in L2 environment	(1)	Duration of immersion	[years]
	(2)	Age of immersion	[years]
	(3)	Distance since immersion	[years]
L2 proficiency	(1)	Proficiency subtest: Grammar	[%]
	(2)	Proficiency subtest: Communication	[%]
	(3)	Proficiency subtest: Production	[%]
	(4)	Proficiency self-evaluation	5-point scale [1: good—5: poor]
Motivation to improve L2 proficiency	(1)	Frustration due to lack of written comprehension	10-point scale [10: high—1: low]
	(2)	Frustration due to lack of oral comprehension	10-point scale [10: high—1: low]
	(3)	Aim of L1 accent avoidance	10-point scale [10: very important—1: not important]
	(4)	Aim to gain automaticity in L2	10-point scale [10: very important—1: not important]
Frequency of daily L2 use	(1)	Frequency of daily L2 use	[%]

### Time-frequency analysis

Time-frequency analyses were performed using the EEGLAB toolbox (version 13.2.2) [90] for MATLAB (version 7.12.0, R2011a). The original sampling rate of 1000Hz was kept throughout the analyses. In order to assess event-related spectral perturbations (ERSP) [100], i.e. event-related shifts in the power spectrum, event-related synchronization (ERS) and desynchronization (ERD) was calculated for target- and saccade-locked segments in the mixed task blocks. ERS and ERD represent the relative power increase (ERS) or decrease (ERD) in a post-stimulus interval relative to a pre-stimulus baseline. A baseline of 500 ms was used in order to obtain at least two oscillatory cycles as a baseline reference even in the lowest frequency involved, i.e. 4 Hz. For target-locked segments a baseline from 800 ms to 300 ms pre-target onset (which is equivalent to the 500 ms baseline before cue onset) and for saccade-locked segments from 1100 ms to 600 ms before saccade onset (which covers a time window that is on average before cue-onset) were chosen. For the frequencies from 4 to 40 Hz, the number of cycles for Morlet wavelets was set to 2 for the lowest frequency but was set to increase with increasing frequencies while allowing to the same degree for an adjustment of the time window (medium between FFT and wavelet analysis (FFT: keeping the time window constant for all frequencies; Wavelet: keeping the number of cycles constant for all frequencies)). Statistical analyses included the factors Saccade task (prosaccade vs. antisaccade) and Group (bilingual vs. monolingual). The analyses conducted for the ERSP data consisted of parametric t-tests (False Discovery Rate (FDR) corrected for multiple comparisons) to test for simple and main effects of these factors and Bootstrapping to test for an interaction between these factors. A significance level of 0.05 was used for all statistical tests and only significant results are reported.

## Results

### Behavioral results

Behavioral data are presented in Table 4 and inferential statistics on behavioral data in Table 5.

**Table 4:** Behavioral data for single and mixed task sessions (‘Saccade task’) and for assessing transition effects (‘Transition’). Error rates (ERR) are given in percentage [%] and saccade latencies (SL) in milliseconds [ms].

	Total		Bilinguals		Monolinguals	
	(*n* = 39)		(*n* = 19)		(*n* = 20)	
	Mean	(SD)	Mean	(SD)	Mean	(SD)
**Saccade task**						
ERR Prosaccades (single) [%]	.92	(2.34)	.47	(.77)	1.35	(3.17)
ERR Antisaccades (single) [%]	13.59	(9.11)	13.47	(7.56)	13.70	(10.57)
ERR Prosaccades (mixed) [%]	1.38	(1.50)	1.47	(1.65)	1.30	(1.38)
ERR Antisaccades (mixed) [%]	27.90	(15.42)	25.95	(13.75)	29.75	(17.00)
SL Prosaccades (single) [ms]	277.44	(21.49)	279.21	(21.28)	275.75	(22.10)
SL Antisaccades (single) [ms]	354.44	(42.78)	360.95	(36.95)	348.25	(47.80)
SL Prosaccades (mixed) [ms]	279.31	(29.62)	282.68	(23.69)	276.10	(34.65)
SL Antisaccades (mixed) [ms]	337.64	(42.68)	343.79	(34.93)	331.80	(49.12)
**Transition**						
ERR PP [%]	0.85	(1.65)	0.97	(1.96)	0.73	(1.33)
ERR AP [%]	2.00	(1.98)	1.95	(2.08)	2.05	(1.94)
ERR AA [%]	25.87	(15.59)	24.25	(14.41)	27.41	(16.87)
ERR PA [%]	29.35	(16.16)	27.13	(14.31)	31.46	(17.85)
SL PP [ms]	276.20	(25.78)	279.36	(22.59)	273.20	(28.75)
SL AP [ms]	288.19	(27.31)	295.78	(27.46)	280.97	(25.78)
SL AA [ms]	336.98	(44.52)	342.68	(34.61)	331.57	(52.58)
SL PA [ms]	340.32	(47.26)	347.78	(40.98)	333.23	(52.60)

AA, Antisaccade repetition; AP, Prosaccade switch; PA, Antisaccade switch; PP, Prosaccade repetition; SD, Standard Deviation.

**Table 5:** Inferential statistics for behavioral data in single and mixed task sessions (‘Saccade task’) and for assessing transition effects (‘Transition’).

Factors	df	Error rates			Saccade latencies		
		*F*	*p*	MSE	*F*	*p*	MSE
**Saccade task**							
Sacc	1,37	118.79	***	125.64	228.60	***	782.81
G	1,37	<1		-	<1		-
Sacc × G	1,37	<1		-	<1		-
Block	1,37	75.96	***	27.86	11.27	**	191.70
Block × G	1,37	<1		-	<1		-
Sacc × Block	1,37	64.18	***	28.86	27.33	***	124.90
Sacc × Block × G	1,37	1.81	>.10	-	<1		-
**Transition**							
Sacc	1,37	115.63	***	230.23	165.38	***	752.04
G	1,37	<1		-	1.15	>.10	-
Sacc × G	1,37	<1		-	<1		-
Transit	1,37	12.16	***	17.06	25.98	***	89.90
Transit × G	1,37	<1		-	3.96	>.05	-
Sacc × Transit	1,37	3.26	>.05	15.97	4.22	*	175.22
Sacc × Transit × G	1,37	<1		-	<1		-

Block, Block type; G, Group; MSE, Mean squared error; Sacc, Saccade task; SD, Standard Deviation; Tran, Transition

* *p <* .05

** *p <* .01

*** *p <* .001.

#### Error rates

The three-way ANOVA including the factors Saccade task, Block type and Group conducted on error rates revealed a main effect of Saccade task (*F*(1, 37) = 118.79, MSE = 125.64, *p* < .001, *η*^2^_p_ = .763), reflecting the higher error rates for antisaccades (20.7 ± 14.4%) than prosaccades (1.1 ± 2.0%). Moreover, there was a main effect of Block type (*F*(1, 37) = 75.96, MSE = 27.86, *p* < .001, *η*^2^_p_ = .672), reflecting the higher error rates in mixed task blocks (14.6 ± 17.1%) than single task blocks (7.3 ± 9.1%). Finally, there was a Saccade task by Block type interaction (*F*(1, 37) = 64.18, MSE = 28.86, *p* < .001, *η*^2^_p_ = .634), indicating that for antisaccades the error rates were significantly higher in mixed task blocks (27.9 ± 15.4%) than in single task blocks (13.6 ± 9.1%; *t*(38) = 8.55, *p* < .001), whereas there was no difference for prosaccades (mixed task blocks: 1.4 ± 1.5%; single task blocks: 0.9 ± 2.3%; *t*(38) = 1.32, *p* = .195), and that the Saccade task effect was larger in mixed task blocks (d = 26.5%, *t*(38) = 10.96, *p* < .001) than in single task blocks (d = 12.7%, *t*(38) = 9.03, *p* < .001).

The three-way ANOVA including the factors Saccade task, Transition and Group to investigate the factor Transition corresponding to the transition effect (i.e. influence of the transition between trial *n*-1 and trial *n*, switch vs. repetition) revealed a main effect of Saccade task (*F*(1, 37) = 115.63, MSE = 230.23, *p* < .001, *η*^2^_p_ = .758), reflecting the higher error rates in antisaccades (27.6 ± 15.4%) compared to prosaccades (1.4 ± 1.5%). Moreover, there was a main effect of Transition (*F*(1, 37) = 12.16, MSE = 17.06, *p* < .001, *η*^2^_p_ = .247), reflecting the higher error rates in switch (15.7 ± 8.3%) compared to repetition trials (13.4 ± 8.0%).

#### Saccade latencies

The three-way ANOVA including the factors Saccade task, Block type and Group conducted on saccade latencies revealed a main effect of Saccade task (*F*(1, 37) = 228.60, MSE = 782.81, *p* < .001, *η*^2^_p_ = .861) reflecting the longer latencies for antisaccades (346 ± 43 ms) than prosaccades (278 ± 26 ms). Moreover, there was a main effect of Block type (*F*(1, 37) = 11.27, MSE = 191.70, *p* < .01, *η*^2^_p_ = .234), indicating on average longer latencies in single task blocks (316 ± 51 ms) than in mixed task blocks (308 ± 47 ms). Finally, there was a Saccade task by Block type interaction (*F*(1, 37) = 27.33, MSE = 124.91, *p* < .001, *η*^2^_p_ = .425) revealing that on average saccade latencies for antisaccades were significantly longer in single task blocks (354 ± 43 ms) than in the mixed ones (338 ± 43 ms; *t*(38) = 5.77, *p* < .001), while there was no difference for the prosaccade latencies between the two blocks (single task blocks: 277 ± 21 ms; mixed task blocks: 279 ± 30 ms; *t*(38) = -0.69, *p* = .496), and that the Saccade task effect was larger in single task blocks (d = 77 ms, *t*(38) = 14.20, *p* < .001) than in mixed task blocks (d = 58 ms, *t*(38) = 14.30, *p* < .001).

In the analysis of saccade latencies in dyads, the three-way ANOVA including the factors Saccade task, Transition and Group revealed a main effect of Saccade task (*F*(1, 37) = 165.38, MSE = 752.04, *p* < .001, *η*^2^_p_ = .817) indicating that the averaged saccade latencies were longer for antisaccade (339 ± 45 ms) than for prosaccade trials (282 ± 27 ms). Moreover, there was a main effect of Transition (*F*(1, 37) = 25.98, MSE = 89.90, *p* < .001, *η*^2^_p_ = .412) reflecting the longer latencies in switch (314 ± 46 ms) compared to repetition trials (306 ± 47 ms). Finally, there was Saccade task by Transition interaction (*F*(1, 37) = 4.22, MSE = 175.22, *p* < .05, *η*^2^_p_ = .102) indicating that there was a significant Transition effect in prosaccades (d = 12 ms, *t*(38) = 5.89, *p* < .001) but not in antisaccades (d = 3 ms, *t*(38) = 1.08, *p* = .289). Moreover, there was a tendency towards a Transition by Group interaction (*F*(1, 37) = 3.96, MSE = 89.90, *p* = .054, *η*^2^_p_ = .097), reflecting a tendency towards a larger Transition effect in bilinguals (d = 11 ms; *t*(18) = 4.08, *p* < .001) than in monolinguals (d = 5 ms; *t*(19) = 3.00, *p* < .01), whereas there was no difference between groups for repetition trials (*t*(37) = 0.80, *p* = .427) or switch trials (*t*(37) = 1.31, *p* = .198).

### ERP results

In the following, first the results of the mixed task blocks and subsequently those of the single task blocks will be presented. Table 6 and Table 7 display an overview of the statistics for lateral and midline electrodes for the two task blocks.

**Table 6:** Analyses of ERP data for the lateral electrodes.

Factors		Cue-locked pos.			N2			P3			PSP			Factors		LPP			LPP		
																Antisaccades			Prosaccades		
		Cue			Target			Target			Saccade					Target			Target		
		150–250			160–200			200–400			-250–50					400–650			400–550		
	df	*F*	*p*	MSE	*F*	*p*	MSE	*F*	*p*	MSE	*F*	*p*	MSE		df	*F*	*p*	MSE	*F*	*p*	MSE
*Mixed task blocks*																					
Sacc	1,37	8.85	**	0.19	12.07	***	0.44	15.56	***	0.70	8.75	**	0.34	Tran	1,37	20.56	***	0.19	10.98	**	0.30
G	1,37	<1		-	<1		-	<1		-	1.18		-	G	1,37	2.01		-	<1		-
Sacc × G	1,37	4.37	*	0.19	<1		-	<1		-	1.90		-	Tran × G	1,37	<1		-	<1		-
Reg	1,37	22.17	***	4.70	5.73	*	16.75	30.91	***	19.34	11.87	***	6.51	Reg	1,37	36.67	***	10.75	54.84	***	12.80
Reg × G	1,37	<1		-	<1		-	<1		-	2.90		-	Reg × G	1,37	<1		-	<1		-
Hem	1,37	<1		-	9.32	**	2.90	<1		-	4.23	*	1.47	Hem	1,37	<1		-	3.76		-
Hem × G	1,37	<1		-	4.14	*	2.90	2.58		-	1.21		-	Hem × G	1,37	1.52		-	<1		-
Sacc × Reg	1,37	1.21		-	<1		-	16.57	***	0.81	9.87	**	0.72	Tran × Reg	1,37	<1		-	6.63	*	0.47
Sacc × Reg × G	1,37	<1		-	3.13		-	1.13		-	2.07		-	Tran × Reg × G	1,37	<1		-	1.04		-
Sacc × Hem	1,37	1.63		-	<1		-	<1		-	2.02		-	Tran × Hem	1,37	<1		-	<1		-
Sacc × Hem × G	1,37	<1		-	<1		-	2.60		-	<1		-	Tran × Hem × G	1,37	<1		-	2.12		-
Reg × Hem	1,37	<1		-	11.08	**	1.20	6.30	*	1.10	<1		-	Reg × Hem	1,37	2.45		-	3.64		-
Reg × Hem × G	1,37	<1		-	<1		-	<1		-	<1		-	Reg × Hem × G	1,37	<1		-	<1		-
Sacc × Reg × Hem	1,37	<1		-	6.40	*	0.21	<1		-	<1		-	Tran × Reg × Hem	1,37	3.05		-	6.51	*	0.13
Sacc × Reg × Hem × G	1,37	<1		-	<1		-	<1		-	<1		-	Tran × Reg × Hem × G	1,37	2.37		-	3.32		-
*Single task blocks*																					
Sacc	1,37	23.44	***	0.22	19.39	***	0.84	18.82	***	0.86	39.95	***	0.97								
G	1,37	<1		-	<1		-	<1		-	<1		-								
Sacc × G	1,37	1.43		-	<1		-	<1		-	1.59		-								
Reg	1,37	7.88	**	4.16	<1		-	28.92	***	15.15	7.09	*	3.50								
Reg × G	1,37	<1		-	<1		-	<1		-	2.04		-								
Hem	1,37	<1		-	16.95	***	1.35	<1		-	<1		-								
Hem × G	1,37	<1		-	5.09	*	1.35	<1		-	2.02		-								
Sacc × Reg	1,37	<1		-	5.75	*	0.82	9.92	**		6.35	*	0.70								
Sacc × Reg × G	1,37	1.59		-	<1		-	1.49		-	<1		-								
Sacc × Hem	1,37	<1		-	<1		-	<1		-	<1		-								
Sacc × Hem × G	1,37	1.76		-	<1		-	2.15		-	<1		-								
Reg × Hem	1,37	<1		-	7.71	**	0.91	3.42		-	<1		-								
Reg × Hem × G	1,37	1.13		-	<1		-	<1		-	<1		-								
Sacc × Reg × Hem	1,37	<1		-	<1		-	<1		-	<1		-								
Sacc × Reg × Hem × G	1,37	<1		-	<1		-	1.20		-	<1		-								

Cue-locked pos., Cue-locked positivity; G, Group; Hem, Hemisphere; LPP, Late parietal positivity; MSE, Mean squared error; PSP, Presaccadic positivity; Reg, Region; Sacc, Saccade task; Tran, Transition

* *p <* .05

** *p <* .01

*** *p <* .001.

**Table 7:** Analyses of ERP data for the midline electrodes (Fz, Cz, Pz).

Factors		Cue-locked pos.			N2			P3			PSP			Factors		LPP			LPP		
																Antisaccades			Prosaccades		
		Cue			Target			Target			Saccade					Target			Target		
		150–250			160–200			200–400			-250–50					400–650			400–550		
	df	*F*	*p*	MSE	*F*	*p*	MSE	*F*	*p*	MSE	*F*	*p*	MSE		df	*F*	*p*	MSE	*F*	*p*	MSE
*Mixed task blocks*																					
Sacc	1,35	7.66	**	0.55	16.97	***	1.71	30.43	***	2.61	25.94	***	0.95	Tran	1,35	20.18	***	0.87	7.84	**	1.16
G	1,35	<1		-				<1		-	1.41		-	G	1,35	<1		-	<1		-
Sacc × G	1,35	5.25	*	0.55	<1		-	<1		-	4.68	*	0.95	Tran × G	1,35	3.33		-	<1		-
Elec	2,70	18.81	***	4.95	22.29	***	19.67	46.92	***	23.91	18.99	***	7.87	Elec	2,70	34.39	***	15.16	45.17	***	17.33
Elec × G	2,70	<1		-	<1		-	1.06		-	3.78	*	7.87	Elec × G	2,70	<1		-	<1		-
Sacc × Elec	2,70	4.03	*	0.38	2.72		-	5.13	*	0.99	11.64	***	0.93	Tran × Elec	2,70	<1		-	1.71		-
Sacc × Elec × G	2,70	<1		-	6.73	**	1.35	4.60	*	0.99	5.66	*	0.93	Tran × Elec × G	2,70	2.43		-	<1		-
*Single task blocks*																					
Sacc	1,35	34.26	***	0.85	24.09	***	1.83	54.58	***	1.94	53.06	***	2.51								
G	1,35	1.27		-	<1		-	<1		-	<1		-								
Sacc × G	1,35	2.29		-	<1		-	1.75		-	3.25		-								
Elec	2,70	16.89	***	4.26	13.15	***	11.11	39.40	***	17.17	15.16	***	3.44								
Elec × G	2,70	<1		-	<1		-	<1		-	2.52		-								
Sacc × Elec	2,70	7.58	**	0.34	3.64	*	1.51	8.32	***	1.07	7.46	**	1.06								
Sacc × Elec × G	2,70	3.07		0.34	<1		-	3.03		-	2.91		-								

Cue-locked pos., Cue-locked positivity; Elec, Electrode; G, Group; LPP, Late parietal positivity; MSE, Mean squared error; PSP, Presaccadic positivity; Sacc, Saccade task; Tran, Transition

* *p <* .05

** *p <* .01

*** *p <* .001.

#### Preparation phase (cue-locked)

**Fig 4:**
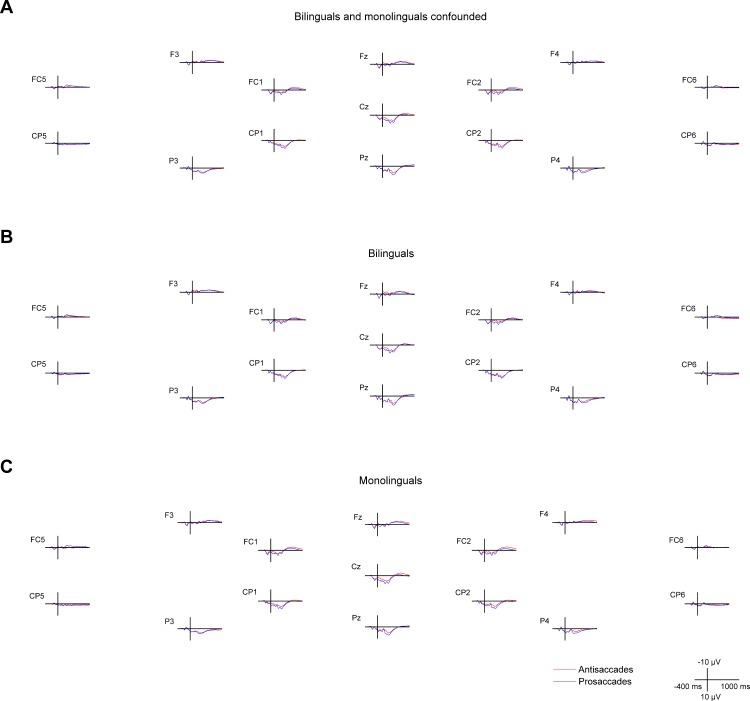
Cue- and target-locked ERPs in the mixed task session on midline and lateral electrodes. The ERPs are presented with -300 ms at cue onset and 0 ms set at target onset, **A** for all bilingual and monolingual participants confounded, **B** for bilingual participants, and **C** for monolingual participants.

**Fig 5:**
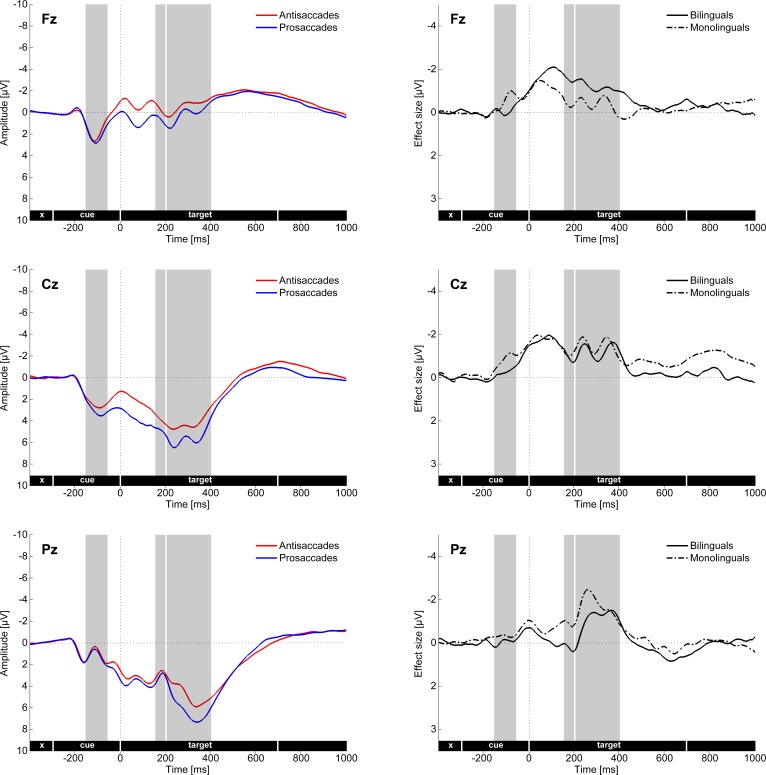
Cue- and target-locked ERPs in the mixed task session on the three midline electrodes (Fz, Cz, Pz). The left panel shows the main effect of Saccade task and the right panel the difference waves (antisaccades minus prosaccades) in the two groups. Grey bars mark the time windows used for investigating the cue-locked positivity effect, as well as the target locked N2 and P3 components.

Cue-locked positivity (cue-locked 150–250 ms): The four-way ANOVA including the factors Saccade task, Region, Hemisphere and Group conducted on lateral electrodes revealed a main effect of Saccade task (*F*(1, 37) = 8.85, MSE = 0.193, *p* < .01, *η*^2^_p_ = .193) reflecting a reduced positivity in the antisaccade compared to the prosaccade condition (Cue-locked positivity effect; Fig 4 and Fig 5). Moreover, there was a significant Saccade task by Group interaction (*F*(1, 37) = 4.37, MSE = 0.193, *p* < .05, *η*^2^_p_ = .106), reflecting that the cue-locked positivity effect was present in the monolingual group (*t*(19) = 3.59, *p* < .01) but not in the bilingual group (*t*(18) = 0.62, *p* = .540), whereas the amplitudes did not differ between the two groups in either antisaccades (*t*(37) = 1.05, *p* = .301) or prosaccades (*t*(37) = -0.06, *p* = .953). The three-way ANOVA including the factors Saccade task, Electrode and Group conducted on midline electrodes (Fz, Cz, Pz) revealed a Saccade task by Electrode interaction (*F*(2, 70) = 4.03, MSE = 0.38, *p* < .05, *η*^2^_p_ = .103; Fig 5), indicating that the cue-locked positivity effect was significantly larger on the Cz compared to the Pz electrode (*F*(1, 35) = 8.03, MSE = 0.43, *p* < .01, *η*^2^_p_ = .187).

#### Implementation phase (target-locked)

N2 (target-locked 160–200 ms): The four-way ANOVA including the factors Saccade task, Region, Hemisphere and Group conducted on lateral electrodes revealed a main effect of Saccade task (*F*(1, 37) = 12.07, MSE = 0.440, *p* < .001, *η*^2^_p_ = .246), reflecting a larger negativity in the antisaccade than in the prosaccade condition (N2 effect; Fig 4 and Fig 5). For midline electrodes (Fz, Cz, Pz), the three-way ANOVA including the factors Saccade task, Electrode and Group revealed a significant Saccade task by Group by Electrode interaction (*F*(2, 70) = 6.73, MSE = 1.35, *p* < .01, *η*^2^_p_ = .161; Fig 5). Post-hoc analyses revealed that there was a significant Saccade task by Group interaction at the Fz electrode (*F*(1, 35) = 5.39, MSE = 1.36, *p* < .05, *η*^2^_p_ = .133), indicating that the N2 effect was present in the bilingual group (*t*(17) = 3.23, *p* < .01) but not in the monolingual group (*t*(18) = 0.35, *p* = .734), whereas the amplitudes did not differ between the two groups in either antisaccades (*t*(35) = -1.34, *p* = .190) or prosaccades (*t*(35) = 0.01, *p* = .992). Moreover, the Saccade task by Group interaction was also significant at the Pz electrode (*F*(1, 35) = 4.83, MSE = 1.13, *p* < .05, *η*^2^_p_ = .121), where the N2 effect was present in the monolingual group (*t*(18) = 2.77, *p* < .05) but not in the bilingual group (*t*(17) = -0.54, *p* = .594), whereas the amplitudes did not differ between the two groups in either antisaccades (*t*(35) = 1.80, *p* = .080) or prosaccades (*t*(35) = 0.46, *p* = .652). Given that the control monitoring-related N2 is characterized as having a fronto-central distribution [77], only the group differences for the N2 effect on the Fz electrode will be discussed subsequently.

P3 (target-locked 200–400 ms): The four-way ANOVA with the factors Saccade task, Region, Hemisphere and Group run on lateral electrodes revealed a significant main effect of Saccade task (*F*(1, 37) = 15.56, MSE = 0.695, *p* < .001, *η*^2^_p_ = .296) reflecting a reduced positivity in the antisaccade compared to the prosaccade condition (P3 effect; Figs 4 and 5). Moreover, there was a Saccade task by Region interaction (*F*(1, 37) = 16.57, MSE = 0.809, *p* < .001, *η*^2^_p_ = .309), indicating that the P3 effect was only significant over the posterior region (*t*(38) = 6.86, *p* < .001) but not over the anterior one (*t*(38) = -0.28, *p* = .781). For the midline electrodes (Fz, Cz, Pz), the three-way ANOVA with the factors Saccade task, Electrode and Group showed a Saccade task by Group by Electrode interaction (*F*(2, 70) = 4.60, MSE = 0.99, *p* < .05, *η*^2^_p_ = .116; Fig 5). However, the post-hoc analyses only revealed a marginal Saccade task by Group interaction at the Pz electrode (*F*(1, 35) = 2.70, MSE = 0.75, *p* = .109, *η*^2^_p_ = .072), indicating a descriptively smaller P3 effect in bilinguals than in monolinguals.

**Fig 6:**
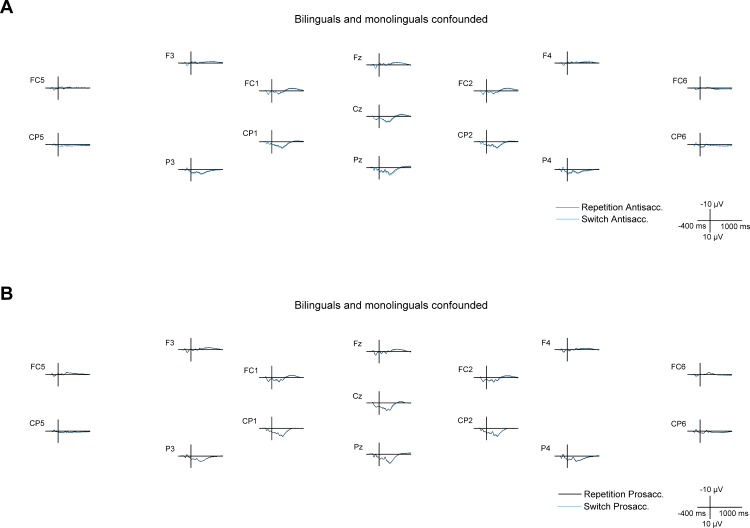
Target-locked ERPs for the Transition types switch and repetition on midline and lateral electrodes. The ERPs are presented **A** for antisaccades, and **B** for prosaccades, for all bilingual and monolingual participants confounded.

**Fig 7:**
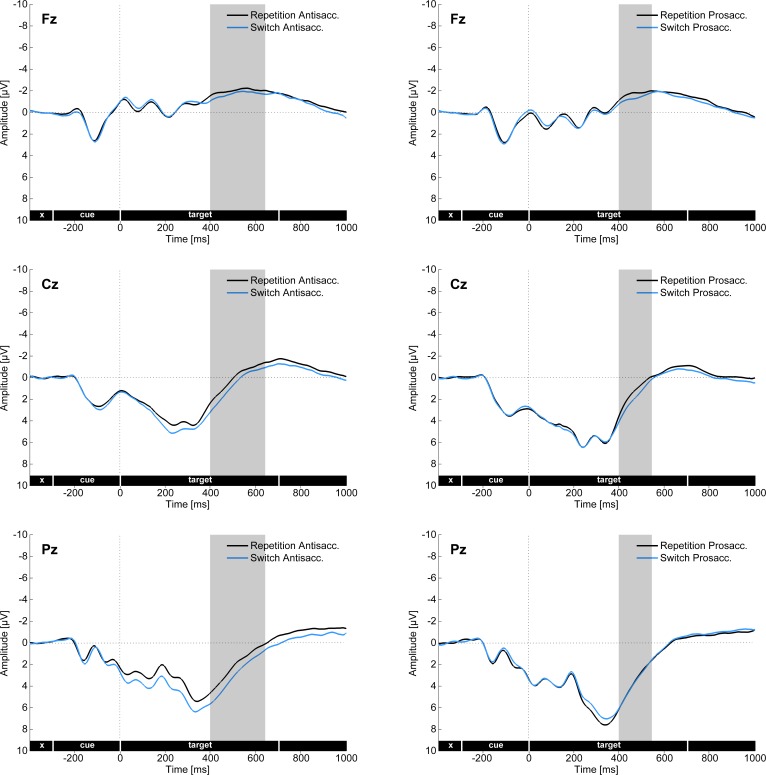
Target-locked ERPs for the Transition types switch and repetition on the three midline electrodes (Fz, Cz, Pz). The left panel shows the ERPs for antisaccade trials and the right panel the ERPs for prosaccade trials, confounded over the two groups. The grey bar marks the time window used for investigating the late parietal positivity (LPP) component.

Late parietal positivity (LPP; target-locked; Antisaccades: 400–650 ms, Prosaccades: 400–550 ms): The LPP effect was tested in the two Saccade tasks separately and after visual inspection, the target-locked time window 400–650 ms was selected for antisaccades while in prosaccades, the time window 400–550 ms was selected. In antisaccades, the four-way ANOVA with the factors Transition, Region, Hemisphere and Group run on lateral electrodes revealed a main effect of Transition (switch vs. repetition; *F*(1, 37) = 20.56, MSE = 0.19, *p* < .001, *η*^2^_p_ = .357), reflecting a larger LPP in the antisaccade switch condition (antisaccade trial preceded by a prosaccade trial) than in antisaccade repetition condition (antisaccade trial preceded by an antisaccade trial; Antisaccade LPP effect; Figs 6 and 7). For prosaccades, the four-way ANOVA with the factors Transition, Region, Hemisphere and Group run on lateral electrodes revealed a main effect of Transition (switch vs. repetition; *F*(1, 37) = 10.98, MSE = 0.30, *p* < .01, *η*^2^_p_ = .229) reflecting a larger LPP in the prosaccade switch condition (prosaccade trial preceded by an antisaccade trial) than in the prosaccade repetition condition (prosaccade trial preceded by a prosaccade trial; Prosaccade LPP effect; Fig 6 and Fig 7). Moreover, there was a Transition by Region interaction (*F*(1, 37) = 6.63, MSE = 0.47, *p* < .05, *η*^2^_p_ = .152) indicating that the prosaccade LPP effect was only present over the anterior region (*t*(38) = -3.86, *p* < .001) but not the posterior one (*t*(38) = -0.06, *p* = .956).

#### Execution phase (saccade-locked)

**Fig 8:**
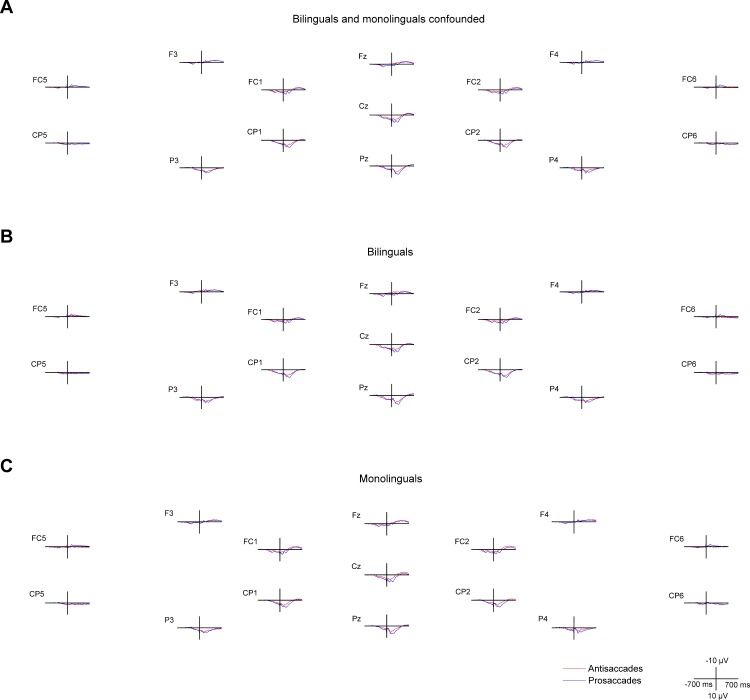
Saccade-locked ERPs in the mixed task session on midline and lateral electrodes. The ERPs are presented **A** for all bilingual and monolingual participants confounded, **B** for bilingual participants, and **C** for monolingual participants.

**Fig 9:**
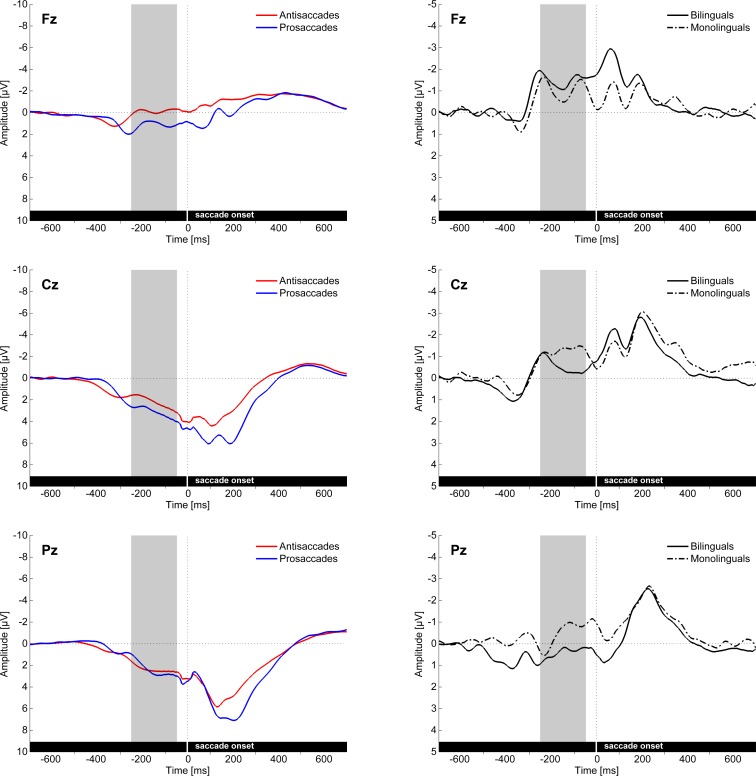
Saccade-locked ERPs in the mixed task session on the three midline electrodes (Fz, Cz, Pz). The left panel shows the main effect of Saccade task and the right panel the difference waves (antisaccades minus prosaccades) in the two groups. The grey bar marks the time window used for investigating the presaccadic positivity (PSP) component.

Presaccadic positivity (PSP; saccade-locked -250 to -50 ms): The four-way ANOVA including the factors Saccade task, Region, Hemisphere and Group conducted on lateral electrodes revealed a main effect of Saccade task (*F*(1, 37) = 8.75, MSE = 0.335, *p* < .01, *η*^2^_p_ = .191), reflecting a reduced PSP in the antisaccade compared to the prosaccade condition (PSP effect; Figs 8 and 9). Moreover, there was a Saccade task by Region interaction (*F*(1, 37) = 9.87, MSE = 0.718, *p* < .01, *η*^2^_p_ = .211) indicating that the PSP effect was present over the anterior region (*t*(38) = 4.05, *p* < .001), but not the posterior one (*t*(38) = -0.90, *p* = .375). The three-way ANOVA including the factors Saccade task, Electrode and Group conducted on midline electrodes (Fz, Cz, Pz) revealed a Saccade task by Group interaction (*F*(1, 35) = 4.68, MSE = 0.952, *p* < .05, *η*^2^_p_ = .118; Fig 9), indicating that the PSP effect was present in the monolingual (*t*(18) = 6.22, *p* < .001) but not in the bilingual group (*t*(17) = 1.78, *p* = .093), whereas the amplitudes did not differ between the two groups in either antisaccades (*t*(35) = 1.87, *p* = .069) or prosaccades (*t*(35) = 0.31, *p* = .759). Moreover, there was a Saccade task by Group by Electrode interaction (*F*(2, 70) = 5.66, MSE = 0.93, *p* < .05, *η*^2^_p_ = .139). Post-hoc analyses revealed a Saccade task by Group interaction at the Cz electrode (*F*(1, 35) = 5.09, MSE = 0.597, *p* < .05, *η*^2^_p_ = .127), indicating that the PSP effect was present in monolinguals (*t*(18) = 6.14, *p* < .001) but not in bilinguals (*t*(17) = 1.41, *p* = .177), and, moreover, that monolinguals showed a marginally significant reduced PSP amplitude compared to bilinguals in antisaccades (*t*(35) = 2.04, *p* = .049), but not in prosaccades (*t*(35) = 0.76, *p* = .453).

#### Single task blocks

**Fig 10:**
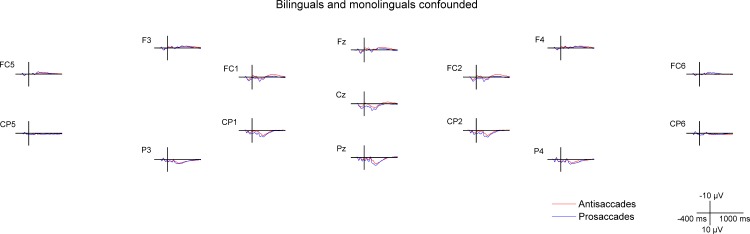
Cue- and target-locked ERPs in single task blocks on midline and lateral electrodes. The ERPs are presented with -300 ms at cue onset and 0 ms set at target onset, for all bilingual and monolingual participants confounded.

**Fig 11:**
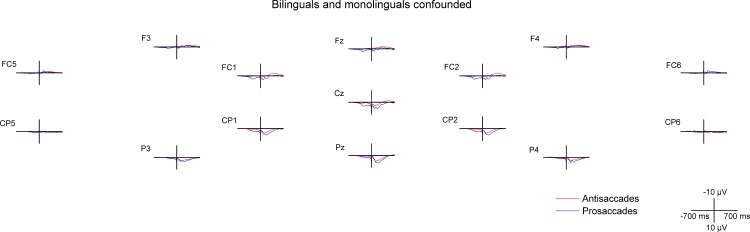
Saccade-locked ERPs in single task blocks on midline and lateral electrodes. The ERPs are presented for all bilingual and monolingual participants confounded.

Moreover, analyses on the single task blocks were conducted because we were interested if a bilingual advantage would show to the same degree if the two saccade tasks were presented in separate blocks. The data and statistics for mixed and single task blocks are presented in Table 6 for lateral electrodes and Table 7 for midline electrodes. ERPs for single task blocks are presented in Fig 10 for cue- and target-locked ERPs and in Fig 11 for saccade-locked ERPs. In the preparation phase, for the cue-locked positivity (cue-locked 150–250 ms), the four-way ANOVA with the factors Saccade task, Region, Hemisphere and Group conducted on lateral electrodes revealed a main effect of Saccade task (*F*(1, 37) = 23.44, MSE = 0.216, *p* < .001, *η*^2^_p_ = .388) reflecting a reduced positivity in the antisaccade compared to the prosaccade condition (Cue-locked positivity effect). The three-way ANOVA with the factors Saccade task, Electrode and Group conducted on midline electrodes (Fz, Cz, Pz) revealed a Saccade task by Electrode interaction (*F*(2, 70) = 7.58, MSE = 0.383, *p* < .01, *η*^2^_p_ = .178), indicating that the cue-locked positivity effect was significantly larger on the Cz as compared to the Fz electrode (*F*(1, 35) = 13.31, MSE = 0.521, *p* < .001, *η*^2^_p_ = .275) and the Pz electrode (*F*(1, 35) = 14.32, MSE = 0.583, *p* < .001, *η*^2^_p_ = .290). In the implementation phase, for the N2 (target-locked 160–200 ms), the four-way ANOVA with the factors Saccade task, Region, Hemisphere and Group conducted on lateral electrodes revealed a main effect of Saccade task (*F*(1, 37) = 19.39, MSE = 0.841, *p* < .001, *η*^2^_p_ = .344), reflecting a larger negativity in the antisaccade than in the prosaccade condition (N2 effect). Moreover, there was a Saccade task by Region interaction (*F*(1, 37) = 5.75, MSE = 0.822, *p* < .05, *η*^2^_p_ = .135), indicating that the N2 effect was present over the posterior region (*t*(38) = 6.64, *p* < .001) but not the anterior one (*t*(38) = 1.19, *p* = .242). For the P3 (target locked 200–400 ms), the four-way ANOVA with the factors Saccade task, Region, Hemisphere and Group run on lateral electrodes showed a significant main effect of Saccade task (*F*(1, 37) = 18.82, MSE = 0.863, *p* < .001, *η*^2^_p_ = .337), reflecting a reduced positivity in the antisaccade compared to the prosaccade condition (P3 effect). Moreover, there was a Saccade task by Region interaction (*F*(1, 37) = 9.92, MSE = 0.838, *p* < .01, *η*^2^_p_ = .211), reflecting that the P3 effect was present over the posterior region (*t*(38) = 6.87, *p* < .001) but not the anterior one (*t*(38) = 0.71, *p* = .482). In the execution phase, for the PSP (saccade-locked -250 to -50 ms prior to saccade onset), the four-way ANOVA with the factors Saccade task, Region, Hemisphere and Group run on lateral electrodes revealed a main effect of Saccade task (*F*(1, 37) = 39.95, MSE = 0.965, *p* < .001, *η*^2^_p_ = .519) reflecting a reduced PSP in the antisaccade compared to the prosaccade condition (PSP effect). Moreover, there was a Saccade task by Region interaction (*F*(1, 37) = 6.35, MSE = 0.703, *p* < .05, *η*^2^_p_ = .146) indicating that the PSP effect was larger over the anterior region (*t*(38) = 5.13, *p* < .001) than the posterior one (*t*(38) = 4.70, *p* < .001). Finally, neither a main effect of Group nor an interaction involving Group was found.

#### Correlation analyses between language background factors and ERP effect sizes

Correlation analyses between language background factors (language switching experience, immersion in L2 environment, L2 proficiency, motivation to improve L2 proficiency, and frequency of daily L2 use) and the ERP effect sizes and electrode sites for which group differences had been found [Cue-locked positivity effect (Cz), N2 effect (Fz), P3 effect (Pz), PSP effect (Cz)] were conducted (Table 8). At the execution phase (saccade-locked), our correlation analyses showed a negative correlation between immersion in the L2 environment and PSP effect size on the Cz electrode (*r*(17) = -.550, *p* < .05). This correlation indicates that the more L2 immersion experience the bilingual participants had, the smaller was their PSP effect size. Moreover, there was a negative correlation between L2 proficiency and PSP effect size on the Cz electrode (*r*(13) = -.742, *p* < .01) indicating that higher L2 proficiency was related to a smaller PSP effect size.

**Table 8:** Pearson correlations between behavioral, ERP and DCM effect sizes on the one hand and language background factors on the other hand, in bilinguals. Data are presented for mixed task blocks.

	Switching		Immersion		L2 prof.		Motivation		L2 freq.
*Behavioral effects*									
ERR	0.263		-0.238		-0.430		-0.091		0.158
SL	-0.047		0.218		-0.264		-0.040		0.198
*ERP effects*									
Cue-loc. pos. eff. (Cz)	0.145		-0.342		-0.344		-0.330		0.370
N2 effect (Fz)	-0.017		-0.454		-0.423		-0.175		0.231
P3 effect (Pz)	0.048		0.074		-0.190		0.033		0.233
PSP effect (Cz)	0.081	**	-0.550	**	-0.742	**	-0.334	**	0.061
*DCM effects*									
ACC → RPFC	0.249		0.277		0.133		-0.251		0.348

Cue-loc. pos. eff., Cue-locked positivity effect; ERR, Error rates; Immersion, Immersion in L2 environment; L2 freq., Frequency of daily L2 use; L2 prof., L2 proficiency; Motivation, Motivation to improve L2 proficiency; SL, Saccade latencies; Switching, Language switching experience; *p*-values for significant correlations (uncorrected for multiple comparisons) are indicated

* *p <* .05

** *p <* .01

*** *p <* .001.

#### Dynamic causal modelling (DCM)

DCM analyses were conducted for pro- and antisaccades issued from mixed task blocks. A highly plausible model was constructed and inverted for each participant, i.e. during the model inversion process, DCM optimizes for each participant the information provided concerning the electromagnetic forward model and the neuronal sources, aiming at minimizing free energy [98]. The DCM included modulatory connections—i.e. which were modelled to vary between the experimental conditions antisaccade vs. prosaccade—between anterior cingulate cortex (ACC), bilateral prefrontal cortex (PFC) and bilateral frontal eye fields (FEF).

#### DCM parameter estimates for modulatory connections

Parameter estimates were extracted for each modulatory effective connection, i.e. connections that were modelled to vary between the experimental conditions antisaccade vs. prosaccade. Independent samples t-tests with Group as a between-subjects factor were run on the parameter estimates of each modulatory connection. Moreover, independent samples t-tests were conducted to examine if the effect was significantly different from 0 in either of the groups. Descriptive and inferential statistics are shown in Table 9. Effective connectivity measures reflect how activity in one brain region influences activity in another region [87]. The independent samples t-test on modulatory parameters revealed a group difference for the RPFC to LFEF modulatory connection (*t*(37) = 2.75, *p* = .01 (FDR-corrected *p* = .096)), indicating that in monolinguals there was a more negative effective connectivity in RPFC to LFEF (-.25 ± .37; *t*(19) = -3.07, *p* = .006 (FDR-corrected *p* = .06)) in antisaccades compared to prosaccades, while this effective connectivity modulation was not significant in bilinguals (.13 ± .50; *t*(18) = 1.18, *p* = .253). Moreover, groupwise analyses showed that in bilinguals there was tendency towards a more negative effective connectivity in ACC to RPFC (-.19 ± .46; *t*(18) = -1.85, *p* = .081 (FDR-corrected *p* = .81)) in antisaccades compared to prosaccades, but not in monolinguals (.03 ± .42; *t*(19) = 0.28, *p* = .78).

**Table 9:** Dynamic causal modelling (DCM): modulatory parameter estimates. Parameter estimates for modulatory connections, i.e. connections that were modelled to vary between the experimental conditions antisaccade vs. prosaccade, are presented for bilingual and monolingual participants.

	Bilinguals					Monolinguals					Effect of Group		
	(*n* = 19)					(*n* = 20)							
	M	(SD)	df	*t*	*p*	M	(SD)	df	*t*	*p*	df	*t*	*p*
ACC**→**LPFC	0.10	(0.52)	18	0.8	0.41	-0.05	(0.41)	19	-0.6	0.57	37	1.0	0.31
ACC**→**RPFC	-0.19	(0.46)	18	-1.8	**0.08**	0.03	(0.42)	19	0.3	0.78	37	-1.6	0.13
LPFC**→**ACC	-0.13	(0.35)	18	-1.7	0.11	-0.08	(0.37)	19	-1.0	0.35	37	-0.5	0.63
RPFC**→**ACC	-0.07	(0.41)	18	-0.7	0.49	-0.15	(0.52)	19	-1.3	0.22	37	0.6	0.58
ACC**→**LFEF	-0.14	(0.50)	18	-1.2	0.24	-0.06	(0.42)	19	-0.6	0.56	37	-0.6	0.57
ACC**→**RFEF	-0.01	(0.31)	18	-0.1	0.90	-0.04	(0.46)	19	-0.4	0.69	37	0.3	0.79
LPFC**→**LFEF	0.12	(0.58)	18	0.9	0.38	0.03	(0.32)	19	0.4	0.71	37	0.6	0.55
LPFC**→**RFEF	-0.19	(0.55)	18	-1.5	0.14	-0.04	(0.49)	19	-0.4	0.70	37	-0.9	0.38
RPFC**→**LFEF	0.13	(0.50)	18	1.2	0.25	-0.25	(0.37)	19	-3.1	**0.01**	37	2.8	**0.01**
RPFC**→**RFEF	-0.09	(0.45)	18	-0.9	0.38	0.02	(0.26)	19	0.4	0.72	37	-1.0	0.34

M, Mean; SD, Standard Deviation. Uncorrected *p* values are given, significant and tendency *p* values are written in bold face.

#### Correlation analyses between language background factors and DCM modulatory connections

Correlation analyses between language background factors and the DCM modulatory connection that were found to be relevant in the bilingual group, i.e. the modulatory effective connection from ACC to RPFC, were conducted for the bilingual group (Table 8). However, there was no significant correlation between any of the five language background factors and the strength of this modulatory effective connection.

### Time-frequency results

**Fig 12:**
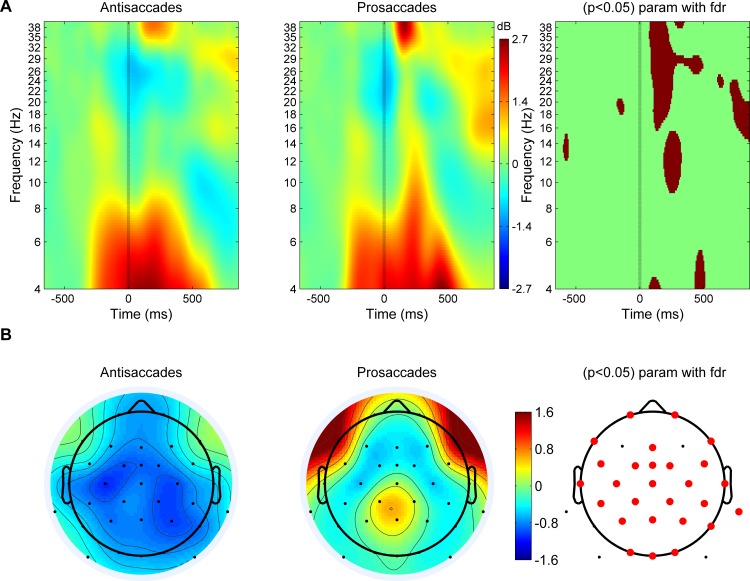
Time-frequency analysis. **A** Event-related spectral perturbations (ERSPs) time-locked to target onset are plotted for the Cz electrode for antisaccades and prosaccades and a panel for significant ERSP differences between Saccade tasks is displayed on the right side. **B** The beta power decrease at around 150 ms after target onset in antisaccades compared to prosaccades over the central and posterior scalp is plotted at frequency 24 Hz. A panel showing the electrodes with a significant ERSP difference between Saccade tasks in red is plotted on the right side.

In order to test for *vector inversion and motor planning*, we tested for target-locked fronto-central and occipital beta (13-26Hz) power changes in anti- compared to prosaccades. There was a significant (*p* < .05, FDR-corrected) power decrease in the antisaccade compared to the prosaccade condition around 150 ms after target onset in the beta band over the vertex and occipital scalp (Fig 12). There was no main effect of or interaction with Group.

## Discussion

In the present study, we investigated the neurodynamics of control processes involved in performing an antisaccade task in two groups differing in language use, i.e. bilinguals and monolinguals. The rationale was to examine whether executive control processes whose efficiency is reinforced by the frequent use of a second language can lead to a benefit in the control of eye movements, i.e. a non-linguistic activity. While the two groups performed similarly at the behavioral level, at the neuronal level clear differences emerged in the ERP measures and dynamic causal modelling (DCM) between bilinguals and monolinguals.

### ERP data

In our study we found a larger target-locked N2 in the antisaccade than in the prosaccade condition, assumed in the literature to reflect conflict monitoring [74–76]. Moreover, the N2 effect was found to be smaller over the Fz in bilinguals than monolinguals, whereas it was larger in bilinguals over the Pz. In an antisaccade task, it is possible that at least two N2 subcomponents can be distinguished, e.g. a conflict monitoring N2 with frontal distribution and a posteriorly distributed N2 reflecting visual attention or target detection [77,101]. The observation of a larger target-locked frontal N2 effect in bilinguals than in monolinguals suggests based on previous studies that this enhancement could be associated with stronger control [102] or stronger conflict monitoring involvement [103]. However, there is currently no unanimous view on the exact functional role of the variation of the N2 amplitude and/or effect size in terms of conflict monitoring capacity [104]. In addition, our ERP data also revealed a group effect for markers of inhibition. A smaller effect size was observed in bilinguals compared to monolinguals for the cue-locked positivity, the target-locked P3 and the saccade-locked presaccadic positivity (PSP), i.e. three ERP markers thought to reflect inhibitory processes / decision to withhold an automatic response [68,83,84]. Moreover, in bilinguals, negative correlations were observed but only between the PSP effect size and (1) L2 proficiency and (2) immersion experience suggesting that these two linguistic factors are good indicators of inhibitory control improvement. Higher L2 proficiency and immersion experience may, due to the continuous need to control over the L1 and the increasingly automatic L2, lead to strengthened inhibitory control. Bilinguals with high compared to those with low second language proficiency have previously been shown to have a behavioral advantage in an oculomotor control task [48], an advantage that had however been attributed to improved performance monitoring rather than inhibitory control. Taken together, the increased N2 effect size in bilinguals, thought to reflect their more efficient conflict monitoring, combined with the reduced effect sizes on markers reflecting inhibitory control, i.e. cue-locked positivity, the target-locked P3 and the saccade-locked presaccadic positivity (PSP), may reflect a dynamic interplay between strengthened conflict monitoring leading to subsequently reduced cost for inhibitory control in realizing the task. While neurophysiological differences between bilinguals and monolinguals were observed for conflict monitoring and inhibition there were no differences on the behavioral level. One reason may be the higher sensitivity of neurophysiological and neuroimaging techniques for disentangling cognitive sub-processes while behavioral measures in many cases allow for assessing a performance that is a product of different sub-processes [105,106]. Finally, no difference between groups was observed for the marker of switching-related activity, i.e. the target-locked late parietal positivity (LPP). That means that bilinguals and monolinguals performed similarly to change from one task to the other in both direction of switch. The type of bilinguals tested in the present study, i.e. late bilinguals immersed in their L1 environment who regularly use their L2, however without frequent switches between the two languages, may have an expertise in conflict monitoring and inhibitory control, but less so in switching-related processes. Hence, further investigation should try to clarify if different types of bilinguals with respect to their language-switching activity show different profiles of control enhancement. To sum up, the present evidence suggests that while bilinguals–at least for the type of bilinguals tested here–do present more efficient neuronal conflict detection and inhibitory control, it was not the case for task switching.

### DCM data

Our study of effective connectivity used a dynamic causal model (DCM) of the executive control network supposed to be involved in eye movement control. The model included modulatory forward connections from the anterior cingulate cortex (ACC) to the prefrontal cortex (PFC) and to the frontal eye fields (FEF), as well as backward from PFC to ACC. For parameter estimates of modulatory connections, differences between the two groups emerged. In DCM, the notion of causality is used in a control theory sense and means that activity in one brain area causes dynamics in another, and that these dynamics cause the observations [87]. In this sense, a positive effective connection indicates that the activity in the source region’s neural population-level activity leads to an increase in the neural population-level activity in the target region, while a negative effective connection indicates that the activity in the source regions leads to a decrease of the activity in the target region. Positive effective connections are thought to reflect an excitatory effect from the source to the target region [107]. Negative effective connections have been interpreted to reflect, either a top-down inhibitory influence from the source to the target region [107,108], or an increase of the response threshold in the target region (leading to a decreased activation in the target region; [108]). Our main interest focused on the effective connections from the anterior cingulate cortex (ACC)–thought to be a core neuronal region involved in conflict monitoring–and the bilateral prefrontal cortex (PFC)–thought to be a crucial region involved in inhibitory control implementation. Group differences were found for the effective connectivity from RPFC to LFEF, with a more negative effective connectivity in antisaccades compared to prosaccades in monolinguals but not in bilinguals. The more negative effective connectivity in RPFC to LFEF in monolinguals but not in bilinguals may reflect a stronger top-down inhibitory influence from PFC to FEF in monolinguals. Moreover, for the connection from ACC to RPFC, we found a more negative effective connectivity in antisaccades compared to prosaccades in bilinguals but not in monolinguals. The more negative effective connectivity from ACC to PFC in bilinguals may reflect a stronger reliance on ACC-directed control (stronger reliance on conflict monitoring) in bilinguals and the more negative effective connectivity from RPFC to LFEF in monolinguals may reflect a stronger reliance on PFC-directed control (stronger involvement of inhibitory control) in monolinguals in performing the antisaccade task. The more negative effective connectivity from ACC to PFC in bilinguals may reflect their strong reliance on highly efficient conflict monitoring in the ACC and its–possibly facilitatory—influence on the subsequent inhibitory control implementation in the PFC, which might indicate that bilinguals resolve conflict in a less costly way than monolinguals. Moreover, the negative connection from ACC to PFC may reflect an inhibitory effect from ACC to PFC activity or in contrast an increase of the response threshold in the PFC, reflected by its reduced activity. Furthermore, this pattern of source activation may also be reflected on the ERP level, on which the increased N2 effect in bilinguals may reflect the stronger reliance on conflict monitoring, which might be causally related to the subsequently smaller effects on markers of inhibitory control, i.e. the cue-locked positivity, the target-locked P3 and the saccade-locked PSP effects. It has previously been shown that individuals with a low (vs. highly) persistent cognitive style show better conflict monitoring ability, and on the neuronal level higher ACC activity and frontal N2 effect size, in performing a Stroop task [103]. Similarly, in the present study, the increased frontal N2 effect size and the ACC-driven neuronal processes might reflect strongly conflict monitoring-driven control in bilingual participants, while in monolinguals inhibitory-driven control appears to be dominant. These group differences in control processes may be linked to the experience with error and uncertainty in second language learning, but further investigation would give precious insight into this question.

To sum up, in bilinguals, the negative effective connectivity from ACC to PFC may indicate a preference in tackling the conflict in an antisaccade task via conflict monitoring. This may hence lead to less costly reactive and inhibitory control implementation, while in monolinguals, the negative effective connectivity from PFC to FEF may indicate that monolinguals preferentially tackle the antisaccade conflict by PFC-based inhibitory control. Moreover, the smaller effect sizes for ERP-markers of inhibition in the present study may also indicate that the reduced cost for inhibitory processes is also due to basically enhanced inhibitory capacities in bilinguals. A key conclusion from the present study is that the investigation of control processes and their neural substrate in isolation might not reveal the most accurate picture but that they should always be considered within their tight interrelation with other control processes. Further investigation should also take into consideration the role of subcortical structures in cognitive and motor control, given their involvement in the antisaccade task [57] as well as in multiple language control [88,89,109], for which neuroimaging techniques such as fMRI or NIRS could provide useful information. Moreover, in EEG studies, a higher number of electrodes would increase the clarity of source and DCM analyses and hence the strength of the effects, which should be taken into consideration in future studies. Moreover, different profiles of bilinguals may show different patterns of control use, hence the study of bilinguals showing a high degree of language switching or mixing as well as bilinguals in early stages of immersion experience may provide further insight on the patterns of neuroplastic control adaptations.

### Domain-general control in multiple language use

The present findings support psycholinguistic theories postulating domain-general control involvement in multiple language control. The sustained coactivation of a bilingual’s languages [12–16] and the bidirectional cross-language influences [19–21] require efficient top-down control in order to allow for the adaptation to speakers of one of the two–or both—languages and for fluid communication. Due to frequent situations of language interference and conflict, bilinguals may in the long run develop more efficient conflict monitoring and inhibitory control in order to prevent and resolve situations of conflict. The observation that ERP and effective connectivity differences between bilinguals and monolinguals are observed in a completely non-linguistic task, i.e. the antisaccade task, is strong evidence that the control processes recruited by bilinguals to control the use of their languages are domain-general and hence shared between different cognitive domains. Moreover, there is evidence that the interplay between conflict monitoring and inhibition processes is optimized in bilinguals in order to reduce the cost in conflict processing.

### Time-frequency data

In order to test for *vector inversion and motor planning*, we tested for target-locked fronto-central and occipital beta (13-26Hz) power changes in anti- compared to prosaccades. There was a significant power decrease in the antisaccade compared to the prosaccade condition around 150 ms after target onset in the beta band over centro-posterior scalp but there was no main effect of or interaction with Group. Note that this effect occurred before saccade onset in prosaccades (279 ± 30 ms) and antisaccades (338 ± 43 ms) and is hence not linked to the muscular activity during motor execution itself. For the change of a motor program, power modulations in the alpha (8–12 Hz) and beta (13–26 Hz) frequency bands have been shown to be of relevance. Alpha and beta oscillations play a role in holding a status quo and for inhibiting movements. Alpha oscillations have been suggested to play a role in the suppression of the excitability in sensory and motor areas, e.g. regulating the receptiveness/readiness in saccadic control network circuits, and to serve a top-down control function for suppressing externally driven saccades in favor of internal goals [110]. Beta oscillations are important for the maintenance of a current motor state, and beta power is highest during holding periods after movements [86]. Beta attenuation however occurs during voluntary movements as well as during preparation and execution of movements, where beta activity is replaced by faster rhythms in the gamma-band (30–100 Hz) [86]. Cordones et al. [85] found stronger fronto-central and occipital beta power decreases in antisaccades compared to nogo trials during the instructive period, which may indicate that these beta power variations do not reflect inhibitory processes but motor planning and preparation. Moreover, beta power decreases have been found in the somatosensory cortex contralateral to the stimulus in an antisaccade task, which has been claimed to reflect somatosensory gating, i.e. increasing the excitability of the cortical region that shows a beta decrease [111]. In accordance with these previous findings, we find beta suppression relatively early, and slightly earlier than proposed in our model (Fig 1), where we suggest vector inversion to be temporally overlapping with conflict monitoring and inhibition processes. This indicates that motor planning processes are also strongly reflected by beta suppression. The occurrence of beta suppression already slightly before conflict monitoring is in accordance with the fact that vector inversion processes are related to preparation and motor planning processes, that take place with the use of the color cue indicating if a vector inversion will have to take place or not, in the present study. Given that the presence or absence of a conflict between movement directions can be prepared, it is hence likely that the motor planning and vector inversion processes start earlier than hypothesized in our model, i.e. already before conflict monitoring, and continue largely in parallel with conflict monitoring as well as with inhibitory processes. To sum up, power variations in the beta band seem to reflect processes of vector inversion and motor planning in the antisaccade task, but these control processes do not seem to differ between bilinguals and monolinguals.

### Limitations

A limitation of the present study is that our groups of participants were not matched for their intelligence quotient (IQ). Some executive control processes have been found to relate to intelligence [112]. Especially, updating of working memory has been found to be correlated with intelligence measures, however less so the here tested inhibiting and shifting functions [112]. Moreover, in the present study, we used a set of 32 channels. A higher number of electrodes would be optimal for source and DCM analyses and hence the strength of our effects. In future studies, these aspects should be taken into consideration in order to increase the power of the experimental design.

## Conclusion

In the present neurophysiological study examining the impact of bilingualism on event-related potentials (ERP), event-related spectral perturbation (ERSP) and the effective connectivity of the underlying neuronal generators (dynamic causal modelling; DCM) in a non-linguistic motor task, we provide evidence for the crucial role of domain-general control involvement in the control over multiple language use. Bilinguals compared to monolinguals show an increased neurophysiological effect for conflict monitoring and reduced neurophysiological effect sizes on markers of inhibitory control. Moreover, there is evidence from dynamic causal modelling that bilinguals rely more strongly on ACC-driven control and monolinguals on PFC-driven control. Finally, L2 proficiency and immersion experience appear to be good predictors of a more efficient inhibitory control.
